# Alzheimer-related decrease in CYFIP2 links amyloid production to tau hyperphosphorylation and memory loss

**DOI:** 10.1093/brain/aww205

**Published:** 2016-08-14

**Authors:** Sachin Suresh Tiwari, Keiko Mizuno, Anshua Ghosh, Wajeeha Aziz, Claire Troakes, Jason Daoud, Vidushi Golash, Wendy Noble, Tibor Hortobágyi, Karl Peter Giese

**Affiliations:** ^1^ 1 Department of Basic and Clinical Neuroscience, Institute of Psychiatry, Psychology and Neuroscience, King’s College London, 125 Coldharbour Lane, London, SE5 9NU, UK; ^2^ 2 Department of Neuropathology, Institute of Pathology, University of Debrecen, 4032 Debrecen, Hungary

**Keywords:** amyloid beta, BACE1, mRNA translation, spatial memory, tau phosphorylation

## Abstract

Characteristic features of Alzheimer’s disease are memory loss, plaques resulting from abnormal processing of amyloid precursor protein (APP), and presence of neurofibrillary tangles and dystrophic neurites containing hyperphosphorylated tau. Currently, it is not known what links these abnormalities together. Cytoplasmic FMR1 interacting protein 2 (CYFIP2) has been suggested to regulate mRNA translation at synapses and this may include local synthesis of APP and alpha-calcium/calmodulin-dependent kinase II, a kinase that can phosphorylate tau. Further, CYFIP2 is part of the Wiskott-Aldrich syndrome protein-family verprolin-homologous protein complex, which has been implicated in actin polymerization at synapses, a process thought to be required for memory formation. Our previous studies on p25 dysregulation put forward the hypothesis that CYFIP2 expression is reduced in Alzheimer’s disease and that this contributes to memory impairment, abnormal APP processing and tau hyperphosphorylation. Here, we tested this hypothesis. First, in post-mortem tissue CYFIP2 expression was reduced by ∼50% in severe Alzheimer’s hippocampus and superior temporal gyrus when normalized to expression of a neuronal or synaptic marker protein. Interestingly, there was also a trend for decreased expression in mild Alzheimer’s disease hippocampus. Second, CYFIP2 expression was reduced in old but not in young Tg2576 mice, a model of familial Alzheimer’s disease. Finally, we tested the direct impact of reduced CYFIP2 expression in heterozygous null mutant mice. We found that in hippocampus this reduced expression causes an increase in APP and β-site amyloid precursor protein cleaving enzyme 1 (BACE1) protein, but not mRNA expression, and elevates production of amyloid-β _42_ . Reduced CYFIP2 expression also increases alpha-calcium/calmodulin-dependent kinase II protein expression, and this is associated with hyperphosphorylation of tau at serine-214. The reduced expression also impairs spine maturity without affecting spine density in apical dendrites of CA1 pyramidal neurons. Furthermore, the reduced expression prevents retention of spatial memory in the water maze. Taken together, our findings indicate that reduced CYFIP2 expression triggers a cascade of change towards Alzheimer’s disease, including amyloid production, tau hyperphosphorylation and memory loss. We therefore suggest that CYFIP2 could be a potential hub for targeting treatment of the disease.

## Teaser

CYFIP2 is thought to regulate mRNA translation at synapses. Tiwari *et al* . reveal reduced CYFIP2 expression in post-mortem Alzheimer’s disease brains, and show that CYFIP2 reduction in mice causes abnormal amyloid production, tau hyperphosphorylation, and spatial memory loss. CYFIP2 could represent a molecular ‘hub’ with potential as a therapeutic target in Alzheimer’s disease.

## Graphical abstract

**Figure:**
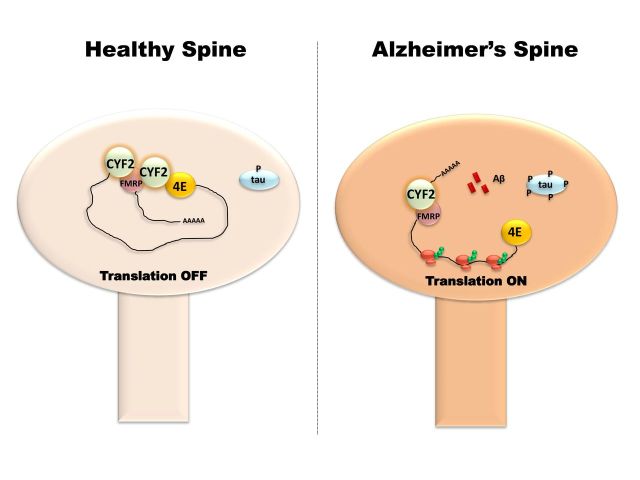
CYFIP2 is thought to regulate mRNA translation at synapses. Tiwari *et al* . reveal reduced CYFIP2 expression in post-mortem Alzheimer’s disease brains, and show that CYFIP2 reduction in mice causes abnormal amyloid production, tau hyperphosphorylation, and spatial memory loss. CYFIP2 could represent a molecular ‘hub’ with potential as a therapeutic target in Alzheimer’s disease.

## Introduction

Alzheimer’s disease is a neurodegenerative disease that causes memory loss. The pathological hallmarks of Alzheimer’s disease are the occurrence of amyloid plaques and neurofibrillary tangles, next to substantial neuronal loss in forebrain ( Braak and Braak, 1991 ; Hardy and Selkoe, 2002 ). Amyloid plaques contain amyloid peptide amyloid-β _42_ that has resulted from abnormal cleavage of amyloid precursor protein (APP). Neurofibrillary tangles contain hyperphosphorylated forms of the microtubule binding protein tau. Further, post-mortem brain analyses have established that synapse loss precedes neuronal loss in Alzheimer’s disease ( Arendt, 2009 ). This synaptic degeneration correlates best with the memory impairment ( deKosky and Scheff, 1990 ; Terry *et al.* , 1991 ; Coleman *et al.* , 2004 ). The initial cause of synaptic degeneration is thought to be formation of soluble amyloid-β _42_ oligomers ( Marcello *et al.* , 2008 ). Amyloid oligomers increase translation of *APP* mRNA at synapses of cultured neurons ( Westmark *et al.* , 2011 ; Sadleir *et al.* , 2014 ), suggesting that increased APP expression contributes to amyloid toxicity ( Westmark, 2013 ). Fragile X mental retardation protein (FMRP, encoded by *FMR1* ) binds to *APP* mRNA and prevents its translation ( Westmark and Malter, 2007 ; Lee *et al.* , 2010 ). Interestingly, FMRP also prevents local translation of mRNA encoding α-isoform of calcium/calmodulin-dependent kinase II (αCaMKII, encoded by *CAMK2A* ) ( Darnell *et al.* , 2011 ), a kinase implicated in tau hyperphosphorylation and Alzheimer’s disease ( Ghosh and Giese, 2015 ). FMRP binds to cytoplasmic FMRP-interacting proteins 1 and 2 (CYFIP1 and CYFIP2) ( Schenck *et al.* , 2001 ). Both of these proteins are expressed at synapses of hippocampal neurons ( Pathania *et al.* , 2014 ). CYFIP1 represses cap-dependent translation of mRNA by interacting with the initiation factor eIF4E ( Napoli *et al.* , 2008 ; Panja *et al.* , 2014 ; Genheden *et al.* , 2015 ). CYFIP2 has an identical eIF4E-binding motif as CYFIP1 ( Napoli *et al.* , 2008 ). In addition to regulating translation of mRNA, CYFIP1 and CYFIP2 are part of the Wiskott-Aldrich syndrome protein-family verprolin-homologous protein (WAVE) complex that regulates actin polymerization at synapses ( Eden *et al.* , 2002 ; De Rubeis *et al.* , 2013 ).

Our previous work showed that in the early stages of Alzheimer’s disease expression of cyclin-dependent kinase 5 (Cdk5) activator p25 is reduced ( Engmann *et al.* , 2011 ). This suggests that Cdk5-mediated phosphorylation of tau may be decreased in Alzheimer’s disease, whereas due to decreased inhibition by Cdk5 glycogen synthase kinase 3β-mediated tau phosphorylation may be increased ( Giese, 2014 ). Further, we showed that p25 overexpression upregulates CYFIP2 expression ( Engmann *et al.* , 2011 ). Therefore, we hypothesized that in early Alzheimer’s disease CYFIP2 expression is reduced and this would contribute to synaptic degeneration. Here, we tested this hypothesis by analysing CYFIP2 and CYFIP1 expression in post-mortem Alzheimer’s disease brain tissue. We found that CYFIP2 expression was reduced by ∼50%. This effect was modelled in mice and we found that reduced CYFIP2 expression leads to increased APP, β-site APP cleaving enzyme 1 (BACE1), and αCaMKII protein expression, abnormal amyloid-β _42_ production, tau hyperphosphorylation at serine-214, alterations in dendritic spine morphology, and memory loss. Therefore, we suggest that CYFIP2 could be one of the key targets for prevention of Alzheimer’s disease.

## Materials and methods

### Post-mortem human brain samples

Human brain samples were obtained from the London Neurodegenerative Diseases Brain Bank, Institute of Psychiatry, Psychology and Neuroscience, King’s College London. They were received in two sets, which were treated separately. The first set contained hippocampal tissue from control subjects, subjects with mild Alzheimer’s disease (Braak stages I–II) and subjects with severe Alzheimer’s disease (Braak stages V–VI) ( *n* = 7 per group), as well as superior temporal gyrus (STG) samples from control and severe Alzheimer’s disease ( *n* = 7 and *n* = 9, respectively). The second set comprised hippocampus and STG samples from control, mild and severe Alzheimer’s disease patients ( *n* = 5 per group). The causes of death were not related to neurodegenerative disease in these control subjects ( Supplementary Table 1 ). All human tissue samples were handled according to the regulations of King’s College London Neurodegenerative Diseases Brain Bank and the Human Tissue Authority.

The frozen samples were lysed at 4°C in RIPA lysis buffer (Santa Cruz Biotechnology, Inc). The RIPA buffer contained 0.1% sodium dodecyl sulphate (SDS), 1% Nonidet P-40, 0.5% sodium deoxycholate and 0.004% sodium azide in Tris-buffered saline (TBS) (pH 7.5). Protease inhibitors cocktail, sodium orthovanadate, and α-toluenesulphonyl fluoride were added to the buffer, diluted to 1:100. The SDS concentration was increased by adding 0.25% SDS in the final volume of buffer. About 100 mg of brain tissue was lysed in 300 μl buffer. Samples were homogenized using a dounce homogenizer (12 strokes, 700 rpm) and centrifuged at 3000 rpm for 10 min. Supernatants were used for western blot analysis.

### Mutant mice

APP _Swe_ (Tg2576) mice, expressing mutant human APP (K670N/M671L) under the control of the hamster prion promoter ( Hsiao *et al.* , 1996 ) were obtained from Taconic farms. Mice were maintained by breeding Tg2576 males in C57BL/6 × SJL F1 genetic background with C57BL/6 × SJL F1 wild-type females, as recommended by the supplier. Mice were genotyped by polymerase chain reaction (PCR) using primer set 5′-CGACTCGACCAGGTTCTGGGT-3′, 5′-ATAACCCCTCCCCCAGCCTAGA-3′. The amplification conditions were as follows. PCR reaction mixture: 1× buffer, 2 mM MgCl _2_ , 0.2 mM dNTP, 0.75 μM APP forward primer, 0.75 μM APP reverse primer, 0.025 μM Taq polymerase (Invitrogen), 2 μl/reaction. PCR program: (i) initialization: 94°C for 3 min; (ii) 35 cycles – denaturation: 94°C for 30 s, annealing: 60°C for 60 s, extension: 72°C for 60 s; (iii) final hold: 4°C. Cortico-hippocampal tissue from 4 month: ( *n* = 3) and 12-month-old ( *n* = 4) Tg2576 mutants as well as wild-type littermates (4 months, *n* = 4; 12 months, *n* = 4) were used for analysis by immunoblotting. Sexes of the animals were balanced.

Cyfip2 ^tm1a(EUCOMM)Wtsi/Wtsi^ mice (EM:05949) were generated in a C57BL/6N background by European Conditional Mouse Mutagenesis Program (EUCOMM). These mutants have an insertion of a promoter-driven neo gene in an intron of the *Cyfip2* gene, which results in a null mutation ( Kumar *et al.* , 2013 ). Therefore, we designated these heterozygous mutants as *Cyfip2*^+/−^ mice. *Cyfip2*^+/−^ mice were maintained in the C57BL/6N background. Mice were genotyped by PCR. The mutants were detected by mutant allele specific 248 bp PCR product (forward CYFIP2 primer 5′-TTCCTTCCTTCCCTTGTCCC-3’, reverse CASR1 primer 5′-TCGTGGTATCGTTATGCGCC-3’) and wild-type mice had only the wild-type allele specific 461 bp PCR product (forward CYFIP2 primer 5′-TTCCTTCCTTCCCTTGTCCC-3’; reverse, CYFIP2 primer 5′-TGCCAGGAGAGACAGTGGTG-3’). The PCR reaction consisted of 1× buffer, 1 mM MgCl _2_ , 0.2 mM dNTP, 10 µM primers and 0.125 μl Taq DNA polymerase (5 U/µl; Invitrogen). The PCR protocol consisted of a 2 min heating at 93°C, followed by 35 cycles of denaturation (30 s, 93°C), annealing (30 s, 56°C) and extension (30 s, 72°C). At the end of the PCR cycle, samples were heated for 10 min at 72°C and held at 4°C until recovered.

Mice were housed on 12 h light:12 h dark cycles with food and water available *ad libitum* . All animal procedures were conducted in accordance with the UK Animals Scientific Procedures Act 1986.

#### Protein preparation from mouse brain

Frozen tissue from Tg2576 mice was homogenized at 100 mg tissue/ml 2× sample buffer (0.5 M Tris-HCl, pH 6.8, 4.4% SDS, 20% glycerol, 2% 2-mercaptoethanol, 0.01% bromophenol blue, and complete mini-protease inhibitor cocktail) (Roche), using a mechanical homogenizer. Following brief sonication, homogenates were centrifuged at 25 000 *g* for 20 min at 4°C, and the supernatant was collected.

For preparation of crude synaptosomes from *Cyfip2*^+/−^ mutants and wild-type littermates, frozen hippocampi were homogenized (10 strokes, 750 rpm) in 20 µl/mg homogenization buffer (0.32 M sucrose, 1 mM NaHCO _3_ , 1 mM MgCl _2_ , 10 mM HEPES pH 7.4). A small volume was collected for analysis of total hippocampal lysates. Following centrifugation at 381 *g* for 10 min, 4°C to remove nuclei and cell debris, the resulting supernatant was centrifuged at 16 089 *g* for 15 min to obtain a crude synaptosomal pellet. Synaptosomal pellets (P2 fraction) were resuspended in 300 µl homogenization buffer for each 50 mg of starting tissue.

A BCA-based protein quantification procedure (Thermo Fisher) was used to determine protein amounts for all mouse samples.

#### Primary neuronal cell culture

Cortical tissues were prepared from embryonic Day 16 mice as described ( Pooler *et al.* , 2012 ). The cells were plated on 6-well dishes coated with poly-D-lysine in neurobasal media (without L-glutamine; with phenol red) containing 2% B27 supplement (Invitrogen), 0.5 mM L-glutamine, and penicillin/streptomycin (50 U/ml / 50 μg/ml, Sigma). After 4 days the cells were cultured in selection media (to remove non-neuronal cells): neurobasal media (without L-glutamine; with phenol red) containing 2% B27 supplement (Invitrogen), 0.5 mM L-glutamine, penicillin/streptomycin, and 3 µM cytosine, β-D-arabinofuranoside. Cells were harvested as described below 21 days after plating.

#### Glial cell culture

Glial cells were isolated from postnatal Day 1–4 (P1–P4) mouse cerebral cortices, as previously described ( Williams and Price, 1995 ) and cultured on poly-D-lysine (25 μg/ml, Sigma) coated T75 flasks at a density of two to three cortices per flask in Dulbecco’s modified Eagle medium (Gibco), supplemented with 10% foetal bovine serum (Biosera) and penicillin/streptomycin (50 U/ml/50 μg/ml, Sigma). Once these cultures reached confluence (12–14 days) they were composed of a base layer of non-dividing astrocytes and an upper layer of dividing microglia and a few oligodendrocytes. For western blot analysis these cells were plated onto 6-well plates; they were harvested from each well using a sterilized scraper and in the presence of 20 µl 2× sample buffer: 0.5 M Tris-HCl, pH 6.8, 4.4% SDS, 20% glycerol, 2% 2-mercaptoethanol, 0.01% bromophenol blue, and 10% mini-protease inhibitor cocktail (Roche).

### Western blot analysis

Protein samples were diluted and boiled at 95°C in Laemmli sample buffer. Comparable protein amounts were separated on 4–15% Criterion™ TGX™ precast gels (Bio-Rad) and then transferred onto a methanol-activated 0.2 μm polyvinylidene fluoride membrane (Bio-Rad). Membranes were blocked at room temperature for 1 h (5% milk in TBST pH 7.5) and then incubated in primary antibody diluted in blocking buffer overnight at 4°C. Primary antibodies were detected using horseradish peroxidase (HRP)-conjugated secondary antibodies (Dako, P0447/8) and chemiluminescent reagent (Thermo Scientific), and signals in the linear range obtained by exposing membranes to X-ray films (Amersham). Prior to probing with other primary antibodies, the membranes were washed in western blot stripping buffer (Santa Cruz Biotechnology). Primary antibodies used were against amyloid-β sequence in APP (Millipore, MABN10, 1:300), APP-CTF sequence in APP (1:10 000; a gift from Prof. C.C. Miller, King’s College London), BACE1 (Gene Tex, GTX62419, 1:5000), αCaMKII (Chemicon, MAB8699, 1:100 000), CYFIP1 (Millipore, 07-531, 1:1000), CYFIP2 (GeneTex, GTX124387, 1:1000), Lamin B1 (Abcam, ab133741, 1:5000), NSE (Millipore, AB951, 1:60 000), synaptophysin (Cell Signaling, 4329, 1:1000), α-synaptotagmin (Sigma, S2177, 1:30 000), phosphoSer214-tau (Abcam, AB10891, 1:250), total tau (Dako, A0024, 1:10 000) and β-tubulin III (Sigma, T8578, 1:1000). Signals were analysed with ImageJ software (NIH).

### Amyloid-β _42_ ELISA

Frozen hippocampal tissue was weighed and manually homogenized in 8× mass of cold 5 M guanidine hydrochloride/50 mM Tris HCl pH 8.0 using a small piston homogenizer. Homogenates were shaken at room temperature for 4 h and 5-fold diluted in cold BSAT-DPBS (5% bovine serum albumin, 0.03% Tween-20, 2.7 mM KCl, 1.5 mM KH _2_ PO _4_ , 136.9 mM NaCl, 8.1 mM Na _2_ HPO _4_ ) supplemented with protease inhibitor cocktail (Sigma). Diluted samples were centrifuged at 27 287 *g* for 30 min and the supernatants (containing GuHCl-soluble amyloid) were collected and stored on ice until use. Supernatants were further 10-fold diluted in standard diluent buffer (supplied) and the assay was carried out according to manufacturer’s instructions (Life Technologies, KMB3441). Samples were measured in duplicates and the entire assay performed twice. As a positive control, tissue from 12-month-old Tg2576 mice was prepared in the same way.

### RNA isolation and quantitative PCR analysis

Total RNA was extracted from hippocampal tissue using TRIzol® (Life Technologies) and purified using RNeasy® spin columns (Qiagen). RNA (1.5 μg) from each sample was reverse transcribed using SuperScript™ II reverse transcriptase (Invitrogen). The cDNA obtained was diluted 10× and stored at −20°C until use. Quantitative RT-PCR experiments were performed using a Chromo4™ Continuous Fluorescence Detector (Bio-Rad DNA Engine®) with KAPA SYBR® FAST Master Mix as a reference dye (KAPA Biosystems). Specific exon primers were designed across introns to unambiguously distinguish spliced cDNA from genomic DNA contamination and purchased from Integrated DNA Technologies. Primers used were APP _Forward_ 5′-ACTCTGTGCCAGCCAATACC-3’; APP _Reverse_ 5′-GAACCTGGTCGAGTGGTCAG-3’; BACE1 _Forward_ 5′-GACCACTCGCTATACACGGG-3’; BACE1 _Reverse_ 5′-TCCTTGCAGTCCATCTTGAGA-3’; CaMKII _Forward_ 5′-TCAGCATCCCAGCCCTAGTT-3’; CaMKII _Reverse_ 5′-AAGGCTCCCTTTCCCAGTTC-3’; HPRT-L _Forward_ 5′-GCTGGTGAAAAGGACCTCT-3’; HPRT-L _Reverse_ 5′-CACAGGACTAGAACACCTGC-3’. Optimum primer and cDNA concentrations were empirically determined; each primer was used at a final concentration of 300 nM and all cDNA was further diluted 50×. The reaction was performed in Thermo-Fast® white 96-well plates capped with Ultra Clear caps (both Thermo Fisher). The reaction mixture was heated at 95°C for 10 min followed by 45 cycles (30 s at 95°C, 30 s at 60°C, 30 s at 72°C), and PCR product levels measured using Opticon Monitor version 3.1.32 (Bio-Rad). Samples were tested in duplicates or triplicates and the entire assay performed twice. mRNA levels were normalized to that of the housekeeping gene *Hprt1* , and the Livak method (or ΔΔC _t_ ) was used for analysis.

### Golgi-Cox staining

Female litters aged 15 weeks of either *Cyfip2*^+/−^ ( *n* = 4) or wild-type genotypes ( *n* = 5) were used to analyse spine density using modified Golgi-Cox staining method as described by the manufacturer (Rapid Golgi; FD NeuroTechnologies). Briefly, brains were removed from skull as quickly as possible, rinsed in Milli-Q® water and stored in Golgi impregnation solution at room temperature in the dark for 2 weeks. Tissue was then transferred into Golgi solution C for 1 week in the dark at room temperature. Coronal sections (150 µm) were obtained using a cryostat and mounted on 2% gelatine-coated glass slides. Sections were rinsed, stained with solution C, dehydrated, cleared of xylene, and were cover slipped and allowed to dry before quantitative analysis. Several pyramidal neurons impregnated with the Golgi solution were readily identified in the dorsal hippocampal region by their characteristic triangular soma shape and numerous dendritic spines. For spine quantification, a 100× oil-immersion objective was used to identify spines in distal dendrites (150 µm away from soma) longer than 10 µm. Straight branches were preferred for *Z* -stack reconstructions to have a clear presentation of spines. More than 50 dendritic segments were analysed for both groups in apical and basal dendrites. Spine densities were calculated as mean numbers of spines per micrometre per dendrite in individual mice per group using ImageJ (NIH). Spines were classified as long-thin, filopodia, or stubby/mushroom, as described ( Harris and Stevens, 1989 ; Harris *et al.* , 1992 ).

### Morris water maze studies

The study with the *Cyfip2*^+/−^ mice and control littermates was performed blind to genotype. Three to 4.5-month-old mutants (10 males) and wild-type mice (13 males) were tested in the hidden-platform version of the water maze. The diameter of the swimming pool was 1.5 m, the platform diameter 0.1 m. After handling the mice for 10 days, 2 min/day, the mice were tested with four trials per day for 10 days. The maximal trial length was 90 s and the intertrial interval was 60 s. Probe trials (60 s) were given at the end of training Day 10 and 5 days after the first probe trial. Four wild-type and three mutants were excluded from analysis due to floating during probe tests. Another group of mice ( *Cyfip2*^+/−^ : eight males, wild-type: eight males) were tested with a visible platform using two trials with a 60 s intertrial interval after hidden platform training for 6 days. Video-tracking and EthoVision XT (Noldus) were used to analyse swimming speed and search strategies.

### Social behaviour and marble burying behaviour

#### Social behaviour

The three-chamber social approach task was performed based on a published method ( Yang *et al.* , 2011 ). Briefly, the subject mouse was habituated to the centre chamber for 10 min and then all three chambers for 10 min. Sociability was tested for 10 min by assessing whether the subject mouse spent more time in the chamber containing a novel mouse (129S2/Sv strain) or in the chamber with an empty cup as novel object. Social novelty was tested for 10 min by assessing whether the subject mouse prefers the chamber containing the first novel mouse or a second novel mouse placed inside the previously empty object cup. Time spent in each chamber was measured using EthoVision XT tracking system (Noldus).

#### Marble burying

The experiments were performed based on a published method ( Deacon, 2006 ). Mice were individually placed for 30 min in animal cages (30 cm × 16 cm) containing 5 cm deep bedding and 18 marbles arranged in three evenly spaced rows of six marbles each. The number of marbles that remained unburied in each 10 min interval was counted. Marbles were counted as buried if they were at least covered two-thirds with bedding.

### Statistical analysis

Unless otherwise specified, data were analysed with *t* -tests.

#### Post-mortem human brain studies

Statistical analysis for individual datasets was performed using unpaired *t* -tests. The pooling of data from two sets of post-mortem tissue samples that were separately obtained was performed using a linear regression model based on the following equation (see also, Tiwari *et al.* , 2015 ):

(1) $${\left(CYFIP2/NSE\right)}_{i}=\;\beta_{0}+\beta_{1}X_{1i}+\beta_{2}X_{2i}+\varepsilon_{i}$$

Where *Χ*_1_*_i_* is the categorical predictor coding for the group difference (e.g. Control versus Severe), and *Χ*_2_*_i_* is the categorical predictor coding for the different experiments (‘1st cohort’ versus ‘2nd cohort’)

This regression model allowed us to pool the CYFIP2 data from the two different sets of post-mortem tissues thereby eliminating potential variability resulting from differences in experimental conditions. Data analysis was performed using SPSS (version 20), which provides the output as a *P* score indicating the overall significance. The contribution and the significance of specific factors of interest, e.g. the disease pathology, are subsequently provided by *t* -test analysis. Hence, this analysis strategy helps to identify the significant change of altered CYFIP2 scores in diseased brain samples when compared to control tissues eliminating any effect induced by the processing of different sample sets. The level of significance for the analysis was 0.05 and the outliers were identified as being out with a threshold of mean ± 4 × standard deviation (SD).

#### Behavioural studies

One-way ANOVA, one-way ANOVA on ranks (if data were not distributed normally), two-way ANOVA with repeated measures, and Student-Newman-Keuls tests were used for statistical analysis as appropriate.

## Results

### Reduced CYFIP2 expression in post-mortem Alzheimer’s disease forebrain

**Figure 1:**
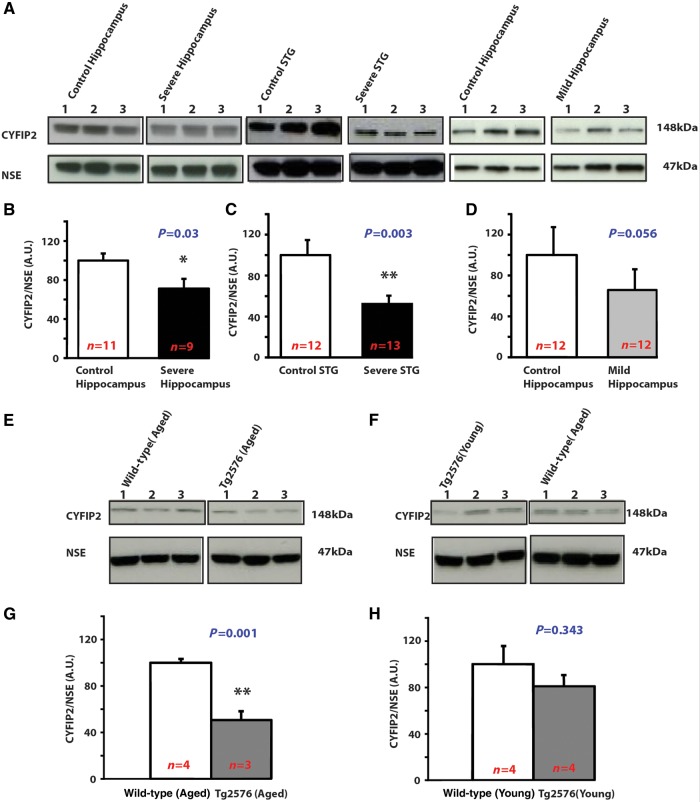
**Reduced CYFIP2 expression in post-mortem Alzheimer’s disease forebrain and in a mouse model of familial Alzheimer’s disease.** ( **A–D** ) CYFIP2 expression is decreased in forebrain of patients with severe Alzheimer’s disease. ( **A** ) Representative western blots. ( **B** ) CYFIP2 expression in hippocampal lysates from patients with severe Alzheimer’s disease ( *n* = 9) and control subjects ( *n* = 11). ( **C** ) CYFIP2 expression in lysates of STG from patients with severe Alzheimer’s disease ( *n* = 13) and control subjects ( *n* = 12). ( **D** ) CYFIP2 expression in hippocampal lysates of patients with mild Alzheimer’s disease ( *n* = 12) and control subjects ( *n* = 12). ( **E–G** ) Age-dependent decrease of CYFIP2 expression in forebrain of Tg2576 mice. ( **E** and **F** ) Representative western blots. ( **G** ) CYFIP2 expression in hippocampal–cortical lysates of 12-month-old wild-type mice ( *n* = 4) and Tg2576 ( *n* = 3). ( **H** ) CYFIP2 expression in hippocampal-cortical lysates of 4-month-old wild-type ( *n* = 4) and Tg2576 mice ( *n* = 4). In all panels CYFIP2 expression was normalized against NSE. Means ± standard error of the mean (SEM) are shown. * *P* < 0.05; ** *P* < 0.01.

To assess whether regulation of local mRNA translation might be affected in Alzheimer’s disease, we investigated CYFIP2 protein levels in forebrain samples of severe disease cases and controls, all of which had short post-mortem delay ( Supplementary Table 1 and Supplementary material ). Antibodies that react specifically with CYFIP2 and neuron-specific enolase (NSE) protein, a neuronal marker ( McAleese *et al.* , 1988 ), were used in western blots ( Supplementary Fig. 1 ). Considering that CYFIP2 is not expressed in glial cells ( Supplementary Fig. 2 ), CYFIP2 expression normalized to NSE expression estimates CYFIP2 expression per neuron at the time of death. We found that CYFIP2 expression was significantly reduced by ∼40% in hippocampus of late Alzheimer’s disease (Braak stages V–VI) in comparison to expression in control subjects ( *t* = −2.33; *P* < 0.05; see Supplementary material ) (Fig. 1A and B).

CYFIP2 protein is expressed at synapses ( Pathania *et al.* , 2014 ). Therefore, reduced CYFIP2 expression in severe Alzheimer’s disease might be due to synapse loss that occurs before neuronal loss ( Arendt, 2009 ). Alternatively, reduced CYFIP2 expression may occur before synapses die in Alzheimer’s disease. We tested for this possibility by normalizing CYFIP2 expression to the presynaptic marker synaptophysin ( Sudhof *et al.* , 1987 ). We found that CYFIP2/synaptophysin expression was significantly decreased by ∼40% in severe Alzheimer’s disease hippocampus ( *t* = 2.43; *P* < 0.05; Supplementary Fig. 3 ), suggesting that CYFIP2 expression declines before synapse loss occurs.

We also studied CYFIP2 expression in the STG, which is affected to a lesser extent and at later stages than the hippocampus in Alzheimer’s disease ( Braak and Braak, 1991 ). CYFIP2 expression was found to be significantly downregulated in severe Alzheimer’s disease STG ( *t* = −3.28, *P* < 0.01; Fig. 1 C), supporting our finding of reduced CYFIP2 expression in the hippocampus.

To investigate if these dysregulations are already present in the early stages of the disease, we analysed hippocampal lysates from patients with mild Alzheimer’s disease (Braak stages I–II). There was a statistical trend towards a reduction of CYFIP2 expression by ∼50% in these cases ( *t* = −2.02, *P* = 0.056; Fig. 1 D). These data suggested that CYFIP2 downregulation may be an early event in Alzheimer’s disease.

CYFIP2 binds to FMRP, a regulator of local mRNA translation ( Schenck *et al.* , 2001 ). Therefore, we studied whether not only CYFIP2 expression but also FMRP expression would be altered in severe Alzheimer’s disease hippocampus ( Supplementary Fig. 4 ). However, we did not find any evidence that FMRP levels are changed in severe Alzheimer’s disease ( *t* = 0.33, *P* = 0.75), suggesting that the reduced CYFIP2 expression is specific.

### Age-dependent reduction of CYFIP2 expression in an Alzheimer’s disease mouse model

We investigated if the reduced CYFIP2 expression observed in post-mortem Alzheimer’s disease brain ( Fig. 1 A–D) can also be found in a mouse model of a familial form of the disease. Tg2576 mice expressing APP with the Swedish mutations do not have amyloid plaques and spatial memory impairment at 4 months of age, whereas they have amyloid plaques and spatial memory deficits at 12 months of age ( Hsiao *et al.* , 1996 ; Stewart *et al.* , 2011 ). We performed a western blot analysis for CYFIP2 expression at these two ages. The comparison revealed significant differences between genotypes. CYFIP2 expression was significantly reduced by 50% in Tg2576 mice in comparison to wild-type mice at 12 months of age ( *t* = −6.56, *P* < 0.01; Fig. 1 E and G). The reduction in CYFIP2 expression is age-dependent, as it was not found for 4-month-old mice ( *t* = −1.03, *P* = 0.34; Fig. 1 F and H). Reduced CYFIP2 expression by ∼50% in 12-month-old Tg2576 mice resembles the reduced CYFIP2 expression in post-mortem Alzheimer’s disease forebrain. Further, it suggests that abnormal APP processing may be sufficient to cause CYFIP2 downregulation.

### CYFIP1 expression is not changed in mild Alzheimer’s disease hippocampus and severe Alzheimer’s disease STG

As CYFIP1 has similar functions *in vitro* to CYFIP2 ( Abekhoukh and Bardoni, 2014 ), we wanted to know if CYFIP1 could be implicated in mild stages of Alzheimer’s disease. We performed western blot analysis with hippocampal protein from patients with mild Alzheimer’s disease and did not observe a downregulation of CYFIP1 expression ( *t* = −1.28, *P* = 0.22; Supplementary Fig. 5A and Supplementary Data ), contrary to the results for CYFIP2 expression ( Fig. 1 D). There was also no change of CYFIP1 expression in STG tissues from patients with severe Alzheimer’s disease ( *t* = −1.15, *P* = 0.26; Supplementary Fig. 5C ), as opposed to CYFIP2 reduction seen in severe Alzheimer’s disease STG ( Fig. 1 C). There appeared to be an upregulation in CYFIP1 levels in severe Alzheimer’s disease hippocampus ( *t* = 3.27, *P* < 0.01; Supplementary Fig. 5D ), contrary to the significant downregulation of CYFIP2 expression ( Fig. 1 B). These results indicate that CYFIP1 and CYFIP2 are regulated differently in Alzheimer’s disease hippocampus.

We further studied whether CYFIP1 expression is altered in old Tg2576 mice, which model early stages of Alzheimer’s disease. As for CYFIP2 ( Fig. 1 E–H), we performed western bot analysis for CYFIP1 expression using the same forebrain tissues of Tg2576 mice and controls. In 4-month-old Tg2576 mouse forebrain there was no change in CYFIP1 expression as compared to wild-type mice ( *t* = 0.97, *P* = 0.37; Supplementary Fig. 5H ). Unexpectedly, we found a downregulation of CYFIP1 expression by ∼60% in 12-month-old Tg2576 mice ( *t* = −5.49, *P* < 0.01; Supplementary Fig. 5G ), instead of an upregulation of CYFIP1 expression observed in post-mortem, severe Alzheimer’s disease hippocampus ( Supplementary Fig. 5D ).

### CYFIP2 regulation is independent of CYFIP1 regulation

**Figure 2:**
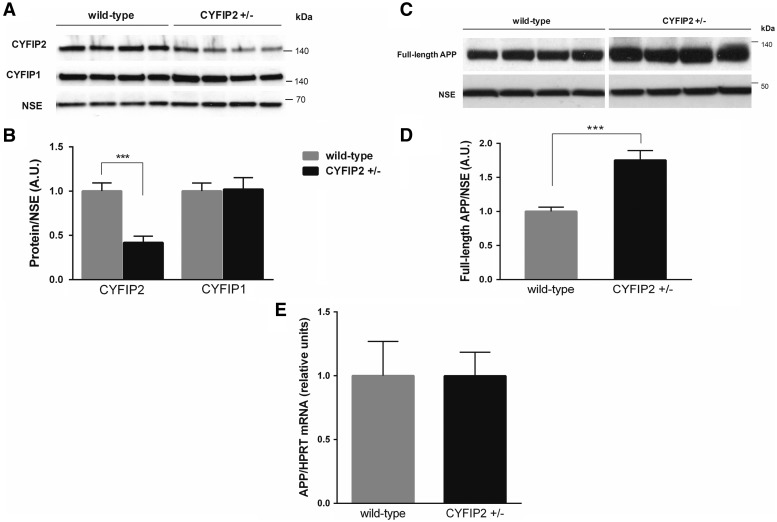
**Reduced CYFIP2 expression does not affect CYFIP1 expression, but leads to increased APP expression at protein but not mRNA level in the hippocampus.** ( **A** ) Representative western blots. ( **B** ) Quantification showed that CYFIP2 expression is reduced by ∼50% in hippocampal synaptosomes of *Cyfip2*^+/−^ mice (black bar; *n* = 7) in comparison to wild-type littermates (grey bar, *n* = 8). CYFIP1 levels are not significantly changed in the same lysates. For quantification CYFIP1 and CYFIP2 expression were normalized to levels of NSE, a neuronal marker. ( **C** ) Representative western blots. ( **D** ) Quantification showed that APP protein expression is significantly increased ∼2-fold in hippocampal synaptosomes of *Cyfip2*^+/−^ mice (black bar) in comparison to wild-type littermates (grey bar; *n* = 8 per genotype). ( **E** ) Quantitative PCR analysis showed that *App* mRNA levels are not elevated in hippocampi of *Cyfip2*^+/−^ mice (black bar) in comparison to wild-type littermates (grey bar) when normalized to the housekeeping gene *Hprt* ( *n* = 8 per genotype). Means ± SEM are shown. *** *P* < 0.001.

We studied the functional impact of reduced CYFIP2 expression in *Cyfip2*^+/−^ mice. We confirmed earlier work ( Kumar *et al.* , 2013 ) that showed that these mutants have reduced CYFIP2 protein expression by ∼50% in hippocampal synaptosomes in comparison to wild-type littermates ( *t* = 4.90, *P* < 0.001; Fig. 2 A and B) (for specificity of hippocampal synaptosomes, see Supplementary Fig. 6 ), and ∼40% in total hippocampal lysates ( Supplementary Fig. 7A and Supplementary Data ). Further, we found that CYFIP1 expression was not altered in hippocampal synaptosomes from *CYFIP2*^+/−^ mutants in comparison to wild-type littermates ( *t* = 0.13, *P* > 0.090; Fig. 2 A and B). Hence, we showed that CYFIP1 and CYFIP2 expression regulations are not dependent on each other.

### Reduced CYFIP2 leads to upregulation of APP protein, but not mRNA expression

At synapses *APP* mRNA is locally translated in an FMRP-dependent manner ( Westmark, 2013 ). As CYFIP2 interacts with FMRP ( Schenck *et al.* , 2001 ), we tested whether reduced CYFIP2 expression impacts on APP protein expression. Western blot analysis with an antibody directed against the C-terminus of APP showed that full-length APP protein expression was significantly elevated ∼2-fold in hippocampal synaptosomes of *Cyfip2*^+/−^ mice in comparison to wild-type littermates ( *t* = 4.84, *P* < 0.001; Fig. 2 C and D). About a 2-fold upregulation of full-length APP protein expression in hippocampal synaptosomes from *Cyfip2*^+/−^ mice was confirmed with an antibody reacting against an epitope in amyloid-β sequence (data not shown). We did not detect a significant increase of full-length APP expression in total hippocampal lysates ( Supplementary Fig. 7A and Supplementary Data ), suggesting that the increase occurs primarily at synapses. We studied whether or not the increased APP expression in *Cyfip2*^+/−^ mice could be due to increased *App* mRNA expression. Quantitative RT-PCR (qPCR) analysis showed that the levels of *App* mRNA in hippocampus did not differ between *Cyfip2*^+/−^ mutants and wild-type littermates ( *t* = 0.007, *P* > 0.99; Fig. 2 E). This result shows that reduced CYFIP2 expression impacts post-transcriptionally on APP expression.

### Reduced CYFIP2 leads to upregulation of BACE1 protein, but not mRNA, and abnormal production of amyloid-β _42_

**Figure 3:**
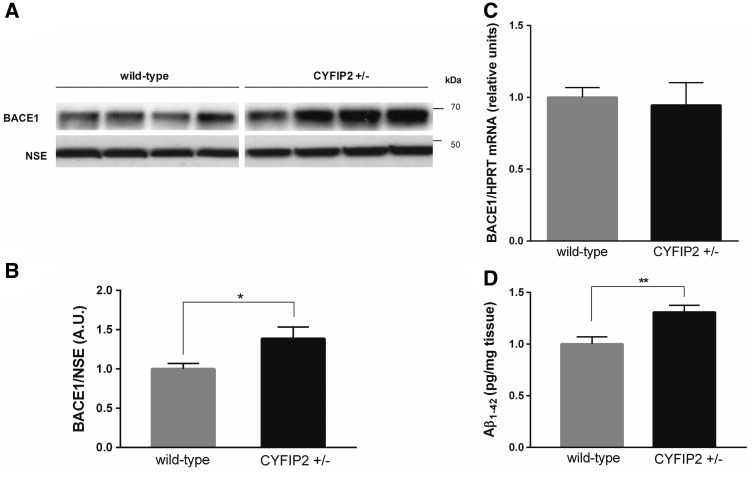
**
Reduced CYFIP2 expression leads to increased BACE1 expression at protein, but not mRNA level, and abnormal amyloid-β _42_ production in the hippocampus.** ( **A** ) Representative western blots. ( **B** ) Quantification showed that BACE1 protein expression is significantly upregulated by ∼40% in hippocampal synaptosomes of *Cyfip2*^+/−^ mice (black bar) in comparison to wild-type littermates (grey bar; *n* = 8 per genotype). ( **C** ) Quantitative PCR analysis showed that *Bace1* mRNA levels are not elevated in hippocampus of *Cyfip2*^+/−^ mice (black bar) in comparison to wild-type littermates (grey bar; *n* = 8 per genotype). ( **D** ) An ELISA on whole hippocampi showed significantly elevated amyloid-β _42_ levels by 30% in *Cyfip2*^+/−^ mutants (black bar; *n* = 4) versus wild-type littermates (grey bar; *n* = 5). Means ± SEM are shown. * *P* < 0.05, ** *P* < 0.01.

Translation of the mRNA encoding β-site APP cleaving enzyme 1 ( *BACE1* ) is increased by amyloid-β _42_ action on cultured neurons ( Sadleir *et al.* , 2014 ; Mamada *et al.* , 2015 ) and in an Alzheimer’s disease mouse model ( Caccamo *et al.* , 2015 ). As the elevated APP protein, but not mRNA, expression in *Cyfip2*^+/−^ mutants ( Fig. 2 C–E) suggested that reduced CYFIP2 expression leads to increased translation of particular mRNAs, we tested whether BACE1 expression was altered. Western blot analysis showed that BACE1 protein expression was significantly elevated in hippocampal synaptosomes by ∼40% in *Cyfip2*^+/−^ mice in comparison to wild-type littermates ( *t* = 2.36, *P* < 0.05; Fig. 3 A and B). This change was not observed in total hippocampal lysates ( Supplementary Fig. 7A and Supplementary Data ), possibly due to high levels of BACE1 expression in non-synaptic fractions (data not shown), suggesting BACE1 expression is mainly elevated at synapses in *Cyfip2*^+/−^ mutants. Quantitative PCR analysis showed that the *Bace1* mRNA expression level was not altered in the hippocampus of *Cyfip2*^+/−^ mutants ( *t* = 0.48, *P* = 0.64; Fig. 3 C). Thus, reduced CYFIP2 expression impacts on the post-transcriptional expression of BACE1. In previous studies higher levels of BACE1 protein expression have been associated with abnormal APP cleavage and higher amyloid-β _42_ production ( Caccamo *et al.* , 2015 ). Therefore, we studied whether amyloid-β _42_ production is elevated in hippocampus of *Cyfip2*^+/−^ mutants. As expected, an ELISA showed that amyloid-β _42_ production was increased by ∼30% in hippocampus of *Cyfip2*^+/−^ mutants in comparison to wild-type littermates ( *t* = 3.18, *P* < 0.01; Fig. 3 D). These findings indicate that reduced CYFIP2 expression affects APP processing.

### Reduced CYFIP2 leads to upregulation of aCaMKII protein, but not mRNA, and increases tau phosphorylation

**Figure 4:**
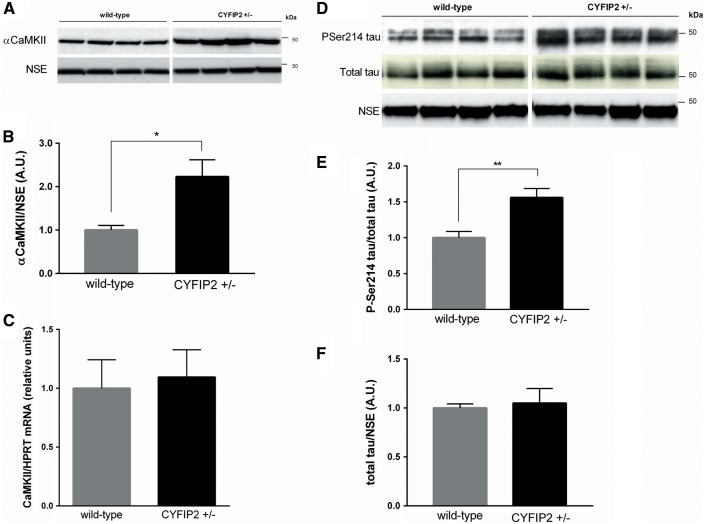
**Reduced CYFIP2 expression leads to increased αCaMKII expression at protein but not mRNA level, and increased tau phosphorylation in the hippocampus.** ( **A** ) Representative western blots. ( **B** ) Quantification showed that αCaMKII protein expression is significantly upregulated ∼2-fold in hippocampal synaptosomes of *Cyfip2*^+/−^ mice (black bar) in comparison to wild-type littermates (grey bar; *n* = 8 per genotype). ( **C** ) Quantitative PCR analysis showed that *Camk2a* /αCaMKII mRNA levels are not elevated in hippocampus of *Cyfip2*^+/−^ mice (black bar) in comparison to wild-type littermates (grey bar; *n* = 8 per genotype). ( **D** ) Representative western blots. ( **E** ) Quantification showed an ∼50% increase in phosphorylation of tau at serine-214 in hippocampal synaptosomes of *Cyfip2*^+/−^ mice (black bar) in comparison to wild-type littermates (grey bar; *n* = 8 per genotype). ( **F** ) Quantification showed that total levels of tau did not differ in hippocampal synaptosomes of *Cyfip2*^+/−^ mice (black bar) in comparison to wild-type littermates (grey bar; *n* = 8 per genotype). Means ± SEM are shown. * *P* < 0.05, ** *P* < 0.01.

Like *App* mRNA, *Camk2a* (αCaMKII) mRNA is locally translated in an FMRP-dependent manner ( Napoli *et al.* , 2008 ). Moreover, recent studies have suggested that increased αCaMKII activity contributes to tau hyperphosphorylation in Alzheimer’s disease ( Ghosh and Giese, 2015 ). Therefore, we studied whether reduced CYFIP2 expression affects αCaMKII expression in the hippocampus. Western blot analysis showed that αCaMKII protein expression was significantly elevated ∼2.5-fold in hippocampal synaptosomes in *Cyfip2*^+/−^ mice in comparison to wild-type littermates ( *t* = 3.05, *P* < 0.05; Fig. 4 A and B). This effect was not observed in total hippocampal lysates ( Supplementary Fig. 7A and Supplementary Data ). This is likely due to a dilution of synaptic signal in total lysates as αCaMKII is also highly expressed in somata and dendrites of hippocampal neurons ( Giese *et al.* , 1998 ). Quantitative PCR analysis showed that the *Camk2a* /αCaMKII mRNA expression level was not altered in the hippocampus of *Cyfip2*^+/−^ mutants ( *t* = 0.39, *P* = 70; Fig. 4 C). This result suggests that CYFIP2 regulates translation of *Camk2a* /αCaMKII mRNA. We tested also whether the increased αCaMKII protein expression correlates with altered tau phosphorylation at serine-214 (S214), a site that is phosphorylated by CaMKII, and one of the key sites that are hyperphosphorylated in Alzheimer’s disease ( Lee *et al.* , 2001 ). The expression of total tau was not altered in *Cyfip2*^+/−^ mutants in comparison to wild-type littermates ( *t* = 0.31, *P* = 076; Fig. 4 D and F). However, phosphorylation of tau at S214 was significantly increased by ∼60% in *Cyfip2*^+/−^ mice in comparison to wild-type littermates ( *t* = 3.63, *P* < 0.01; Fig. 4 D and E).

### Reduced CYFIP2 affects spine morphology on apical, but not basal, dendrites of hippocampal CA1 pyramidal neurons

**Figure 5:**
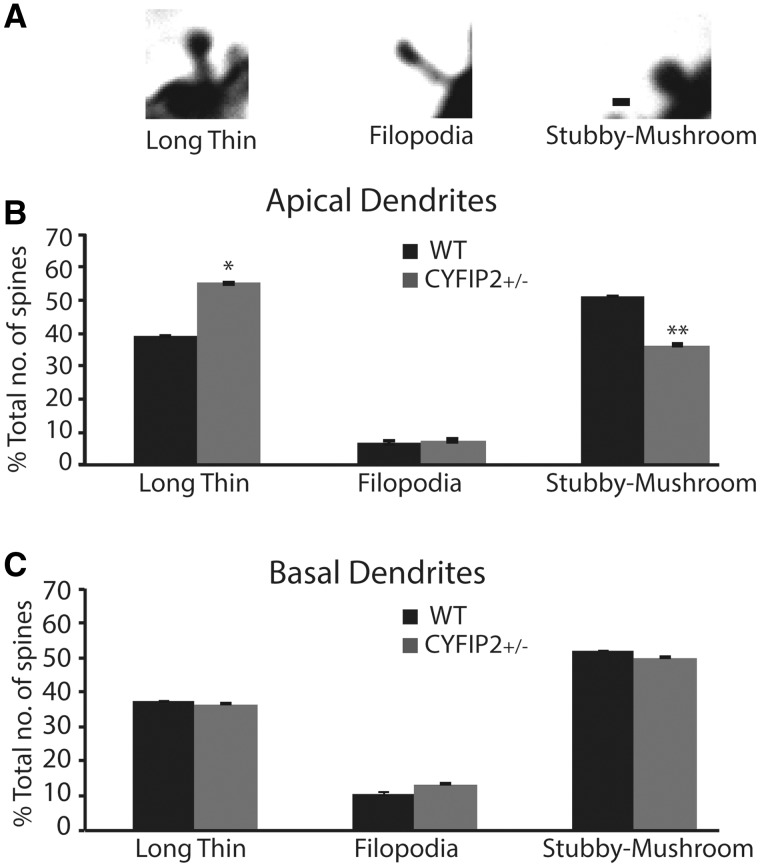
**Reduced CYFIP2 expression leads to altered spine morphology in apical dendrites of CA1 pyramidal neurons.** ( **A** ) Spines were classified as long-thin, filopodia, or stubby/mushroom, according to criteria set by Harris *et al.* (1992) . Spines were imaged under bright-field microscopy using a 100× oil-immersion objective. Scale bar = 0.5 µm. ( **B** ) Spines classified as a proportion of total number of spines for apical dendritic segments of dorsal CA1 pyramidal neurons. Wild-type (WT) controls (black bars, *n* = 5) were compared to *Cyfip2*^+/−^ mutants (grey bars, *n* = 4) for long-thin spines (39% versus 55%), filopodia (6.7% versus 7.5%), and stubby/mushroom (51% versus 36%). *Cyfip2*^+/−^ mutants have significantly more long thin spines, but less stubby-mushroom spines than wild-type mice. ( **C** ) Spines classified as a proportion of total number of spines for basal dendritic segments of dorsal CA1 pyramidal neurons. Wild-type controls (black bars, *n* = 5) were compared to *Cyfip2*^+/−^ mutants (grey bars, *n* = 4) for long-thin, filopodia, and stubby/mushroom spines. No difference was found between the genotypes. Means ± SEM are shown. * *P* < 0.05; ** *P* < 0.01.

CYFIP2 not only binds to FMRP, it is also part of the WAVE complex ( Eden *et al.* , 2002 ), which regulates actin polymerization. As CYFIP1 is also part of the WAVE complex and because reduced CYFIP1 expression affects dendritic spine morphology of hippocampal CA1 pyramidal neurons ( De Rubeis *et al.* , 2013 ; Pathania *et al.* , 2014 ), we studied whether reduced CYFIP2 expression would also alter dendritic spines. Using Golgi-Cox staining, we analysed dendritic spines on apical and basal segments of CA1 pyramidal neurons of *Cyfip2*^+/−^ mutants and wild-type littermates ( Supplementary Fig. 8 ). There was no significant difference in spine density between the genotypes for apical distal segments ( *t* = 0.095, *P* = 0.93) or for basal distal segments ( *t* = 0.10, *P* = 0.92) ( Supplementary Fig. 8C and Supplementary Data ). Next, we categorized the spines into three classes: long-thin, filopodia and stubby-mushroom spines according to widely used criteria ( Harris and Stevens, 1989 ; Harris *et al.* , 1992 ) ( Fig. 5 A). Comparison of these spine types on apical distal dendrites revealed a significant difference between genotypes ( Fig. 5 B). Compared to wild-type littermates, *Cyfip2*^+/−^ mutants had a greater proportion of long-thin spines (55% for mutants versus 39% for wild-types; *t* = 3.45, *P* < 0.05) and a smaller proportion of stubby/mushroom spines (36% for mutants versus 51% for wild-types; *t* = 3.51, *P* < 0.01). The proportion of filopodia, however, remained constant between the two groups ( *t* = 0.61, *P* = 0.56). In contrast with the differences in apical spine morphology between genotypes, we found that the abundance of spine types on basal dendrites was not altered in *Cyfip2*^+/−^ mutants (long-thin, *t* = 0.34, *P* = 0.75; filopodia, *t* = 0.99, *P* = 0.37; stubby/mushroom, *t* = 0.51, *P* = 0.63; Fig. 5 C). These findings show that reduced CYFIP2 expression affects spine morphology only in particular dendritic segments of CA1 pyramidal neurons.

### Reduced CYFIP2 impairs retention of spatial memory

**Figure 6:**
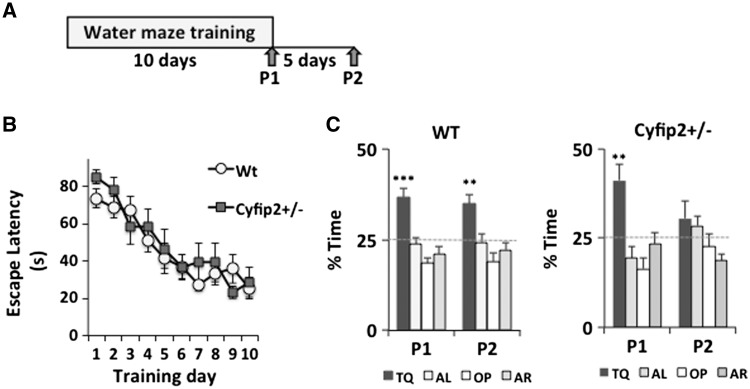
**Reduced CYFIP2 expression impairs the maintenance of spatial memory.** ( **A** ) The experimental design for the hidden-platform training in the Morris water maze. Mice were trained with four trials per day for 10 days. Probe trials were given at the end of training Days 10 (P1) and 5 after the end of training (P2). ( **B** ) The time to reach the hidden platform did not differ between wild-type ( *n* = 9) and *Cyfip2*^+/−^ mutant mice ( *n* = 7). Both groups improved with training. ( **C** ) Both wild-type and *Cyfip2*^+/−^ mutants searched selectively target quadrant in a probe trial given immediately after training Day 10 (P1). However, *Cyfip2*^+/−^ mutants did not search selectively in the second probe trial after 5 days of training (P2), in contrast to wild-type mice. Thus, the mutants cannot maintain spatial memory. TQ = target quadrant; OP = opposite quadrant; AL = adjacent left quadrant; AR = adjacent right quadrant. Means ± SEM; *** *P* < 0.001, ** *P* < 0.01.

Hippocampus-dependent spatial memory is affected in the early stages of Alzheimer’s disease *.* To investigate whether reduced CYFIP2 expression affects spatial memory formation, we studied the *Cyfip2*^+/−^ mutants and wild-type littermates in the hidden platform version of the water maze ( Fig. 6 ). The mice were trained with four trials per day for 10 days. The genotypes did not differ in latency to locate the platform [two-way ANOVA with repeated measures; effect of genotype *F* (1,14) = 0.29, *P* = 0.60; effect of training *F* (9,126) = 18.9, *P* < 0.001; genotype × training interaction *F* (9,126) = 0.91, *P* = 0.52] ( Fig. 6 B). Studies with a separate cohort of mice indicated that *Cyfip2*^+/−^ mutants had normal visible platform learning (time to reach platform for wild-type mice, 9.6 ± 1.5 s; for mutants, 11.1 ± 2.7 s; one-way ANOVA on ranks H = 0.025, *P* = 0.88. This indicates that the *Cyfip2*^+/−^ mutants were not impaired in swimming abilities, motivation, and vision. To assess hippocampus-dependent spatial memory, probe trials were performed. A probe trial given at the end of training Day 10 (P1) showed that *Cyfip2*^+/−^ mutants formed normal spatial memory after training ( Fig. 6 C). However, a second probe trial given 5 days after first probe test (P2) revealed that *Cyfip2*^+/−^ mutants were impaired in retention of spatial memory, in contrast with wild-type littermates ( Fig. 6 C). During these probe trials the average swimming speed did not significantly differ between genotypes [P1: wild-type mice, 21.7 ± 2.0 cm/s; mutants, 24.3 ± 2.7 cm/s; one-way ANOVA with genotype as variable, *F* (1,14) = 4.58, *P* = 0.051; P2: wild-type, 22.1 ± 1.60 cm/s; mutants, 24.1 ± 2.3 cm/s; one-way ANOVA with genotype as variable, *F* (1,14) = 3.90, *P* = 0.068]. Analysis of search time in the four quadrants during probe trial P1 showed that *Cyfip2*^+/−^ mutants and wild-type littermates searched selectively [one-way ANOVA with analysis of the quadrant as variable; mutants, *F* (3,24) = 9.65, *P* < 0.001; wild-type mice, *F* (3,32) = 17.3, *P* < 0.001] ( Fig. 6 C). The mutants and wild-type mice spent more time searching in the target quadrant than in any other quadrant (Student-Newman-Keuls test; mutants, *P* < 0.001 target quadrant versus opposite quadrant and adjacent left quadrant, *P* = 0.002 target quadrant versus adjacent right quadrant; wild-type, *P* < 0.001 target quadrant versus opposite quadrant, adjacent left quadrant, adjacent right quadrant). On the other hand, during probe trial P2 the *Cyfip2*^+/−^ mutants searched randomly, spending similar times in all quadrants [one-way ANOVA with quadrant as variable, *F* (3,24) = 2.55, *P* = 0.08], in contrast with wild-type littermates [one-way ANOVA with quadrant as variable, *F* (3,32) = 8.47, *P* < 0.001]. The wild-type mice spent more time searching in the target quadrant than in any other quadrant (Student-Newman-Keuls test; *P* < 0.001 target quadrant versus opposite quadrant, *P* = 0.002 target quadrant versus adjacent right quadrant, *P* = 0.004 target quadrant versus adjacent left quadrant). Taken together, these results indicate that *Cyfip2*^+/−^ mutants are able to form spatial memories, but they cannot maintain these memories.

### CYFIP2 downregulation does not cause abnormal social and repetitive behaviour

In humans heterozygous microdeletion of chromosome 15, which includes the *CYFIP1* gene, causes neurobehavioural disturbances including autism ( Abekhoukh and Bardoni, 2014 ; Cox and Butler, 2015 ). However, human genetic studies have not found a link between mutations of the *CYFIP2* gene and autism. Further, CYFIP2 is expressed much later during cortical development in comparison with CYFIP1 ( Bonaccorso *et al.* , 2015 ). We studied whether reduced CYFIP2 expression in *Cyfip2*^+/−^ mice affects social behaviour in the three-chamber task and repetitive behaviour in the marble burrowing task ( Supplementary Fig. 9 ), two behaviours that are impaired in mouse models of autism ( Silverman *et al.* , 2010 ; Santini *et al.* , 2013 ). We found that *Cyfip2*^+/−^ mice did not significantly differ from wild-type littermates in these tasks. Thus, reduced CYFIP2 expression might not cause any neurodevelopmental abnormalities that lead to autism-like behaviours.

## Discussion

Here, we show that expression of CYFIP2 protein is substantially reduced in Alzheimer’s disease forebrain and our functional studies in mice suggest that this CYFIP2 downregulation links together amyloid production, tau hyperphosphorylation and spatial memory loss.

Our previous studies on p25 dysregulation suggested that CYFIP2 expression could be reduced in Alzheimer’s disease ( Engmann *et al.* , 2011 ). Here, post-mortem expression analysis shows that this is the case. To assure that CYFIP2 expression is not simply reduced due to neuronal and synaptic loss in Alzheimer’s disease, we normalized CYFIP2 levels to expression of a neuronal housekeeping protein as well as to a synaptic vesicle protein. The decreased, normalized CYFIP2 expression in post-mortem Alzheimer’s disease forebrain indicates that CYFIP2 levels decline before neurons and synapses die. We suggest that CYFIP2 expression decreases already in the early stages of Alzheimer’s disease, as we found an ∼2-fold reduction in mild Alzheimer’s disease hippocampus (Braak stages I–II). Consistent with this idea, we found that CYFIP2 protein expression is reduced in an APP transgenic mouse line, the Tg2576 mouse, which models early Alzheimer’s disease ( Hsiao *et al.* , 1996 ; Stewart *et al.* , 2011 ). This finding also suggests that abnormal APP processing may be sufficient to decrease CYFIP2 expression. Follow-up investigations will be needed to characterize the mechanism by which abnormal APP processing downregulates CYFIP2 expression.

We also investigated CYFIP1 protein expression in the Alzheimer’s disease forebrain, as CYFIP1 and CYFIP2 are thought to have similar functions *in vitro* , although these two proteins do not compensate for each other in the haploinsufficient state in mice or humans ( De Rubeis *et al.* , 2013 ; Pathania *et al.* , 2014 ; Cox and Butler, 2015 ). In contrast to CYFIP2 expression, the level of CYFIP1 expression is not changed in post-mortem Alzheimer’s disease brain, with the exception that CYFIP1 expression is elevated in severe Alzheimer’s disease hippocampus. This particular increase in CYFIP1 expression in severe Alzheimer’s disease hippocampus, but not in severe Alzheimer’s disease superior temporal gyrus, may represent a late wave of further neurodegeneration in the disease, or alternatively a compensatory mechanism contributing to the survival of some hippocampal neurons in the disease ( West *et al.* , 1994 ). It is also important to note that a study of large rare copy number variants in Alzheimer’s disease among Caribbean Hispanics identified a nominal association between Alzheimer’s disease and a chromosomal duplication that includes the *CYFIP1* gene ( Ghani *et al.* , 2012 ). This duplication was found in 10 Alzheimer’s disease cases (2.6%) versus three controls (0.8%).

Synapse loss and the resulting impact on synaptophysin expression is uneven within the Alzheimer’s disease hippocampus ( Heinonen *et al.* , 1995 ; Honer, 2003 ). Reduced synaptophysin expression is pronounced in area CA1, where most neuronal loss occurs ( West *et al.* , 1994 ). Additionally, reduced synaptophysin expression is thought to occur particularly in neurons with neurofibrillary tangles ( Coleman *et al.* , 2004 ; Arendt, 2009 ). However, in a western blot analysis where synaptophysin expression was normalized to expression of a neuronal marker protein we and others did not detect reduced synaptophysin expression in Alzheimer’s disease hippocampal homogenates ( Davidsson *et al.* , 1998 ; Tiwari *et al.* , 2015 ). This is in agreement with a recent study showing that in mammalian models of tauopathy there is little evidence that synaptophysin expression is lost when normalized to NSE expression in brain regions affected by significant neurodegeneration ( Bondulich *et al.* , 2016 ). This suggests that synaptophysin levels are not reduced in most neurons that have not yet died. A similar observation was made for synapsin I expression in severe Alzheimer’s disease cortex ( Kurbatskaya *et al.* , 2016 ), whereas expression of other synaptic markers is altered ( Honer, 2003 ; Kurbatskaya *et al.* , 2016 ).

We studied the functional impact of reduced CYFIP2 expression in heterozygous null mutant mice. We found that reduced CYFIP2 expression increases APP and αCaMKII expression in synapses at the post-transcriptional level, similar to previous findings with CYFIP1 ( Napoli *et al.* , 2008 ). FMRP binds to *APP* and *CAMK2A* /αCaMKII mRNAs and suppresses their translation ( Westmark and Malter, 2007 ; Lee *et al.* , 2010 ; Darnell *et al.* , 2011 ). Further, CYFIP2 binds to FMRP ( Schenck *et al.* , 2001 ) and has a binding motif for the translational initiator eIF4E ( Napoli *et al.* , 2008 ). Taken together, this suggests that CYFIP2 normally suppresses translation of *APP* and *CAMK2A* /αCaMKII mRNAs and that reduction of CYFIP2 expression is sufficient to increase translation of these mRNAs.

Studies with various models of Alzheimer’s disease have suggested that there is a feed-forward mechanism of amyloid-β _42_ production that involves elevated translation of *APP* mRNA ( Westmark *et al.* , 2011 ; Westmark, 2013 ; Sadleir *et al.* , 2014 ; Caccamo *et al.* , 2015 ). In this scenario, increased APP protein expression would provide more material for abnormal APP cleavage resulting in further amyloid-β _42_ production. The amyloid-β _42_ -induced elevation of *APP* mRNA translation does not involve phosphorylation of the translation initiation factor eIF2α ( Sadleir *et al.* , 2014 ), and is not well understood at the mechanistic level. Our work suggests that reduction of CYFIP2 expression is an important step in upregulating *APP* mRNA translation in a feed-forward amyloid-β _42_ production process. Additionally, recent studies have shown that amyloid-β _42_ oligomers elevate BACE1 expression post-translationally, suggesting that not only increased APP protein expression, but also enhanced BACE1 protein expression may be part of a feed-forward mechanism for amyloid-β _42_ production ( Sadleir and Vassar, 2012 ; Mamada *et al.* , 2015 ). Interestingly, we found that reduced CYFIP2 expression increases BACE1 expression at the post-transcriptional level. As *BACE1* mRNA is not regulated by FMRP ( Buffington *et al.* , 2014 ), it is possible that this increased expression is secondary to elevated amyloid-β _42_ production resulting from increased APP protein expression when CYFIP2 expression is reduced. Thus, reduction of CYFIP2 expression may be an essential step for increasing APP and BACE1 protein expression for the feed-forward production of amyloid-β _42_ .

A fundamental question in Alzheimer’s disease research is how abnormal APP processing causes tau hyperphosphorylation, which is a prerequisite for neurofibrillary tangle formation. Our work suggests that reduced CYFIP2 expression links amyloid production with tau hyperphosphorylation. Reduced CYFIP2 expression is suggested to increase translation of mRNA encoding *CAMK2A* /αCaMKII, resulting in increased CaMKII activity, which leads to hyperphosphorylation of tau ( Sengupta *et al.* , 1998 ; Wang *et al.* , 2007 ). Tau is phosphorylated at many different sites and in this study we only investigated phosphorylation at S214, a site that is hyperphosphorylated in Alzheimer’s disease ( Lee *et al.* , 2001 ). Follow-up studies are needed to study the impact of reduced CYFIP2 expression on tau phosphorylation in much greater detail.

Our studies in mice have revealed that reduction of CYFIP2 expression leads to a characteristic spatial memory phenotype: spatial memory acquisition is not impaired but spatial memory cannot be retained and is lost within a few days in comparison to normal wild-type mice. To our knowledge there is only one other mutant mouse with a similar spatial memory phenotype, mice lacking the prion-like translational regulator CPEB3 ( Fioriti *et al.* , 2015 ). Thus, it is conceivable that CYFIP2 and CPEB3 regulate the same mRNA translation, which is needed for memory storage. Alternatively, reduced WAVE function may account for the spatial memory loss in CYFIP2 mutant mice, as the mutants have more immature spines and less mature (‘memory’) spines ( Bailey *et al.* , 2015 ). In any case, it is intriguing that reduced CYFIP2 expression links together spatial memory loss with amyloid production and tau hyperphosphorylation, three features and hallmarks of Alzheimer’s disease.

Recently, another laboratory has studied the phenotype of a newly generated *Cyfip2* heterozygous null mutant mouse line ( Han *et al.* , 2015 ). These authors also showed that reducing CYFIP2 expression does not impact on CYFIP1 expression, but they suggested that CYFIP2 does not regulate APP and αCaMKII protein expression. One possible explanation for the discrepancy with our findings is that Han *et al.* (2015) studied APP and αCaMKII expression in cortical, and not hippocampal, neurons. In cortical neurons CYFIP2 expression is higher than in hippocampal neurons ( Han *et al.* , 2015 ) and a higher level of residual CYFIP2 expression may suppress a phenotype. However, Han *et al.* (2015) found a dendritic spine phenotype in cortical but not in hippocampal neurons in their CYFIP2 mutant mouse line. Therefore, it is conceivable that differences in genetic background account for the phenotypic differences. Our *Cyfip2* heterozygotes were maintained on a C57BL/6N genetic background where the residual CYFIP2 has a S968F point mutation that reduces the half-life of CYFIP2 ( Kumar *et al.* , 2013 ), whereas Han *et al.* (2015) used *Cyfip2* heterozygotes in a C57BL/6J genetic background. Applied to Alzheimer’s disease this could suggest that genetic modifiers may suppress the impact of reduced CYFIP2 expression on amyloid production, tau hyperphosphorylation and spatial memory loss in some patients.

In conclusion, to our knowledge this is the first report modelling a protein expression change detected in post-mortem Alzheimer’s disease brain that causes a combination of amyloid production, tau hyperphosphorylation and spatial memory loss, i.e. key features of the disease. This suggests that there are molecular ‘hubs’ that contribute to various aspects of Alzheimer’s disease, and that reduced CYFIP2 expression is one of these. Such ‘hubs’ could be ideal targets for pharmacological treatment where a multitude of neurodegenerative processes may be rectified.

## Supplementary Material

Supplementary Data

## Abbreviations

FMRP: fragile X mental retardation protein
NSE: neuronal specific enolase
STG: superior temporal gyrus
