# Supplementary material for: Alzheimer-related decrease in CYFIP2 links amyloid production to tau hyperphosphorylation and memory loss
Source: Brain. 2016 Aug 14;139(10):2751–65. doi: 10.1093/brain/aww205 (PMC5035822; doi:10.1093/brain/aww205)
Supplement: Supplementary Data [file aww205_supplementary_data.zip › brain-2016-00583-File017.pdf]

Table 1. Details of post-mortem brain tissues. PMD refers to post-mortem delay

Hippocampus –

Control-Mild AD study

S.No	Pathological state	Sex	Age(Years)	PMD(Hours)
1	Control	M	81	18
2	Control	F	92	17
3	Control	M	78	10
4	Control	M	85	16
5	Control	F	76	28
6	Control	M	65	24
7	Control	M	86	6
8	Control	F	72	24
9	Control	F	55	24
10	Control	F	80	31
11	Control	F	71	30
12	Control	M	77	29
13	Mild AD	M	81	12
14	Mild AD	F	92	9
15	Mild AD	F	80	3
16	Mild AD	F	55	12
17	Mild AD	F	81	17

18	Mild AD	F	81	16.5
19	Mild AD	F	82	13
20	Mild AD	M	64	16
21	Mild AD	F	83	24
22	Mild AD	M	81	3
23	Mild AD	M	90	5.5
24	Mild AD	F	94	21

Control-Severe AD study

S.No	Pathological state	Sex	Age(Years)	PMD(Hours)
1	Control	M	81	18
2	Control	F	92	17
3	Control	M	78	10
4	Control	M	85	16
5	Control	F	76	28
6	Control	M	65	24
7	Control	M	86	6
8	Control	F	72	24
9	Control	F	55	24
10	Control	F	80	31
11	Control	F	71	30

12	Control	M	77	29
13	Severe AD	M	64	23
14	Severe AD	F	68	11
15	Severe AD	M	80	15
16	Severe AD	F	69	16
17	Severe AD	M	77	10
18	Severe AD	F	69	16.3
19	Severe AD	F	79	24
20	Severe AD	F	71	21
21	Severe AD	F	82	4.5
22	Severe AD	F	80	4.3
23	Severe AD	F	88	19
24	Severe AD	M	75	17

Superior Temporal Gyrus -

S.No	Pathological state	Sex	Age(Years)	PMD(Hours)
1	Control	F	55	24
2	Control	M	55	24
3	Control	M	65	24
4	Control	M	69	24
5	Control	M	86	6

6	Control	M	65	24
7	Control	M	71	5
8	Control	M	81	18
9	Control	F	92	17
10	Control	M	78	10
11	Control	M	85	16
12	Control	F	76	28
13	Severe AD	F	69	16.3
14	Severe AD	F	71	21
15	Severe AD	F	80	4
16	Severe AD	F	81	24
17	Severe AD	F	82	4.5
18	Severe AD	F	82	12
19	Severe AD	F	88	19
20	Severe AD	F	91	23
21	Severe AD	M	75	17
22	Severe AD	M	64	23
23	Severe AD	F	68	11
24	Severe AD	M	80	15
25	Severe AD	F	69	16
26	Severe AD	M	77	10
