# Supplementary material for: Alzheimer-related decrease in CYFIP2 links amyloid production to tau hyperphosphorylation and memory loss
Source: Brain. 2016 Aug 14;139(10):2751–65. doi: 10.1093/brain/aww205 (PMC5035822; doi:10.1093/brain/aww205)
Supplement: Supplementary Data [file aww205_supplementary_data.zip › brain-2016-00583-File018.pdf]

Synaptic regulator CYFIP2 is decreased in Alzheimer's disease leading to amyloid production, tau hyperphosphorylation, and spatial memory loss in a transgenic mouse model

Sachin Suresh Tiwari[1*], Keiko Mizuno[1*], Anshua Ghosh[1*], Wajeeha Aziz[1], Claire Troakes[1], Jason Daoud[1], Vidushi Golash[1], Wendy Noble[1], Tibor Hortobágyi[1,2], Karl Peter Giese[1]

Supplementary information for statistical analysis of post-mortem samples

Since the data used in this project involved pooling the data from two different sample sets, and hence utilized the regression analysis model for statistical studies, it was not appropriate to perform a correlation study between different parameters and protein levels observed in post-mortem tissues. Hence, we adopted the strategy of comparing the significant difference between the age, post-mortem delay (PMD) and gender (nonparametric analysis) of patient samples grouped into different pathological state of disease– control, mild, severe AD. An analysis for pH couldn't be performed as for samples pH data were not recorded or analysed.

Hippocampus AD samples

A) Effect of Gender – Pearson's chi-square test was performed to discover the relationship between the categorical variable (gender and pathological state). The p=0.63 shows that there was no statistically significant association between gender and pathological state.

B) Effect of Age – One-way ANOVA showed no changes in the age between control, mild and severe AD groups, $F_{2,33}$=0.96, p=0.39.

C) Effect of PMD – One-way ANOVA showed an association between PMD and the pathological state of patients, $F_{2,23}$=4.81, p=0.015. Tukey's posthoc test revealed that PMD was not significantly different between the control and severe pathological state group (p=0.09). It was also not different between the mild and severe pathological state group (p=0.69). However, the PMD between control and mild pathological state showed significant difference (p=0.014). The post-mortem delay in control (21.4±2.3 h) was significantly higher than mild state AD patient hippocampus (12.7±1.9 h).

Comparison of CYFIP2/NSE control subject vs. severe AD hippocampus (Fig. 1B)

The coefficient of determination for the model was R^2 = 0.315 and overall model significance was $F_{2,17}$=3.91, p<0.05 with β_1= -0.056 and β_2 = -0.095. For control vs. severe AD, t= -2.33, P<0.05. The sample size for severe AD was n=9 as three samples were eliminated from the analysis, one being an outlier and for two samples no signal was obtained on the western blots. The sample size for control subjects was n=11 as one sample was eliminated for being an outlier.

Model =

CYFIP2/NSE = 0.580-0.056(old/new)-0.095(ctrl/sev)+ e

Comparison of CYFIP2/NSE control subject vs. mild AD hippocampus (Fig. 1D)

The coefficient of determination for the model was R^2 = 0.774 and overall regression model significance was $F_{2,21}$=36.0, p<0.001 with β_1= 1.286 and β_2 = -0.310. A resulting t-test revealed that there is only a trend of a difference for CYFIP2/NSE expression in control versus mild AD (t= -2.02, p=0.056).

Model =

CYFIP2/NSE = 1.718 + 1.286(old/new)-0.310(ctrl/mild)+ e

STG AD samples

A) Effect of Gender - Pearson's chi-square test was performed to discover the relationship between the categorical variable (Gender and pathological state). The p=0.018, showed a statistically significant association between the individuals gender and its pathological state.

B) <u>Effect of Age</u> – Independent sample two tailed t test showed there was no significant difference in the age between the control and severe STG groups (t= -0.96, p=0.35).

C) <u>Effect of Postmortem delay</u> – Independent sample two tailed t test showed there was no significant difference in the post-mortem delay between the control and STG groups (t=1.05, p=0.306).

Gender showed significant association with the pathological state of STG. However, since none of the studied proteins have been reported to have sex linkages, the possibility of gender differences having an impact on results is negligible. Unfortunately, an exact correlation study cannot be performed with our scores as scores were pooled from different experimental sets. Age and PMD were not significantly different between control and severe AD STG.

Comparison of CYFIP2/NSE control subject vs. severe AD STG (Fig. 1C)

The coefficient of determination for the model was R^2 = 0.488 and overall regression model significance was $F_{2,22}$=10.5, p=0.001 with β_1= 0.146 and β_2 = -0.152. For control vs. severe AD, t= -3.28, P<0.01.

Model =
CYFIP2/NSE = 0.533+0.146(old/new)-0.152(ctrl/sev)+ e
