# Supplementary material for: Alzheimer-related decrease in CYFIP2 links amyloid production to tau hyperphosphorylation and memory loss
Source: Brain. 2016 Aug 14;139(10):2751–65. doi: 10.1093/brain/aww205 (PMC5035822; doi:10.1093/brain/aww205)
Supplement: Supplementary Data [file aww205_supplementary_data.zip › brain-2016-00583-File020.pdf]

Supplementary Figure S1. Specific binding of anti-CYFIP2 and anti-NSE antibodies to post-mortem Alzheimer's disease forebrain proteins. In western blots anti-CYFIP2 and anti-NSE antibodies showed a specific band at the correct molecular weight.

Supplementary Figure S2. CYFIP2 is not expressed in glial cells. Lysates from cultured glial cell cultures (first five lanes from left) along with lysates from mouse cortical neuronal culture (lanes 6-9) and human hippocampal lysates (lanes 10 and 11) were probed with anti-CYFIP2 antibodies. The blot was stripped and probed with anti-actin antibodies to control for protein loading. No CYFIP2 signal was detected in the lanes with glial cell culture.

Supplementary Figure S3. CYFIP2 expression normalized to the synaptic marker synaptophysin is decreased in Alzheimer's disease hippocampus, suggesting that CYFIP2 is reduced before synapses die. **(A)** CYFIP2 expression in hippocampal lysates from severe Alzheimer's disease patients (*n* =6) and control subjects (*n* =7). **(B)** Representative western blots. CYFIP2 protein expression was normalized against synaptophysin expression. Means ± s.e.m. are shown. *, P <0.05.

Supplementary Figure S4. FMRP levels are not changed in severe Alzheimer's disease hippocampus. (A) FMRP expression levels in the hippocampus of control subjects (*n* = 6) and severe Alzheimer's disease patients (*n* =5). **(B)** Representative western blots. FMRP protein expression was normalized against NSE. Means ± s.e.m. are shown.

Supplementary Figure S5. CYFIP1 expression is unchanged in mild Alzheimer's disease post-mortem forebrain and is not modelled in a mouse model of familial Alzheimer's disease. (A) Representative western blots from post-mortem brain study. **(B)** CYFIP1 expression in hippocampal lysates of mild Alzheimer's disease (*n* =12) and control subjects (*n* =12). **(C)** CYFIP1 expression in lysates of superior temporal gyrus (STG) from severe Alzheimer's disease (*n* =13) and control brain (*n* =12). **(D)** CYFIP1 expression in hippocampal lysates from severe Alzheimer's disease (*n* =12) and control brain (*n* =12). CYFIP1 protein expression was normalized against

NSE. **(E, *F*)** Representative western blots from Tg2576 study. **(G)** CYFIP1 expression in hippocampal –cortical lysates of 12 month-old wild-type mice (*n* =4) and Tg2576 (*n* =3). **(H)** CYFIP1 expression in hippocampal-cortical lysates of 4 month-old wild-type (*n* =4) and Tg2576 mice (*n* =4). In all panels CYFIP1 expression was normalized against neuron-specific enolase (NSE). Means ± s.e.m. are shown. *, *P* <0.05; **, *P* <0.01.

Supplementary Figure S6. Purity of synaptosomal preparations from mouse hippocampus. The crude synaptosomal fraction (P2) did not contain contamination from the nuclear fraction (P1) as determined by levels of the nuclear envelope marker Lamin B1. Further, the P2 fraction contained only very low levels of β-tubulin III, which was abundant in the cytosolic fraction (S2). The pre-synaptic marker protein α-synaptotagmin was enriched in P2 and not detectable in S2.

Supplementary Figure S7. Protein expression changes in CYFIP2$^{+/-}$ mice in total hippocampal lysates. (A) Representative western blots of total hippocampal lysates from wild-type and CYFIP2$^{+/-}$ mice for CYFIP2, full-length APP, BACE1, αCaMKII and NSE. **(B)** Quantification (wild-type: *n* =8, CYFIP2$^{+/-}$: *n* =7) showed an approximately 40% reduction of CYFIP2 in these lysates (t=3.68, *P* <0.01). No changes were found in the expression of APP (t=1.64, *P* =0.13), BACE1 (t=1.55, *P* =0.15) and αCaMKII (t=0.59, *P* = 0.57). Means ± s.e.m. are shown. **, *P* <0.01.

Supplementary Figure S8. Reduced CYFIP2 expression does not alter spine density in apical and basal dendrites of CA1 pyramidal neurons. (A) Golgi-Cox staining of hippocampal dorsal CA1 (dCA1) area viewed using 5x objective under bright-field microscopy. Scale bar, 200 µm. CA1 pyramidal cell with apical and basal dendrites at higher magnification. Scale bar, 50 µm. Apical and basal dendrites of CA1 pyramidal cell. Images were taken for spine density analysis from the distal part of apical and basal dendrites (150 µm away from soma). **(B)** Image shows an apical and basal dendritic segment taken using a 100x oil-immersion objective from a wild-type animal and a similar example from a Cyfip2$^{+/-}$ mutant mouse. 67 and 47 dendritic segments were analysed for wild-type and mutant mice, respectively. Scale bar, 5 µm. **(C)** Spine density (Means

± SEM) on apical dendrites in dCA1 for wild-type controls (black bars, *n* =5) and Cyfip2$^{+/-}$ mutant mice (grey bars, *n* =4). Only segments ≥10 μm were analysed, and spine density was quantified. **(D)** Spine density (Means ± SEM) on basal dendrites in dCA1 for wild-type controls (black bars, *n* =5) and Cyfip2$^{+/-}$ mutant mice (grey bars, *n* =4).

Figure S9. Reduced CYFIP2 expression does not cause autism-like behavioural phenotypes. **(A)** Sociability test: Cyfip2$^{+/-}$ and wild-type littermates spent more time in the chamber containing a novel mouse (black bars) than in the chamber containing a novel object (diagonal lines). **(B)** Social novelty test: Cyfip2$^{+/-}$ and wild-type littermates spent more time in the chamber containing a novel mouse (black bars) than in the chamber containing a familiar mouse (diagonal lines). **(C)** Marble burying test: Cyfip2$^{+/-}$ and wild-type littermates buried a similar number of marbles. Means ± s.e.m.; ***P< 0.001, *P<0.05.
