# Supplementary material for: Alzheimer-related decrease in CYFIP2 links amyloid production to tau hyperphosphorylation and memory loss
Source: Brain. 2016 Aug 14;139(10):2751–65. doi: 10.1093/brain/aww205 (PMC5035822; doi:10.1093/brain/aww205)
Supplement: Supplementary Data [file aww205_supplementary_data.zip › Supplementary Figure 2.pdf]

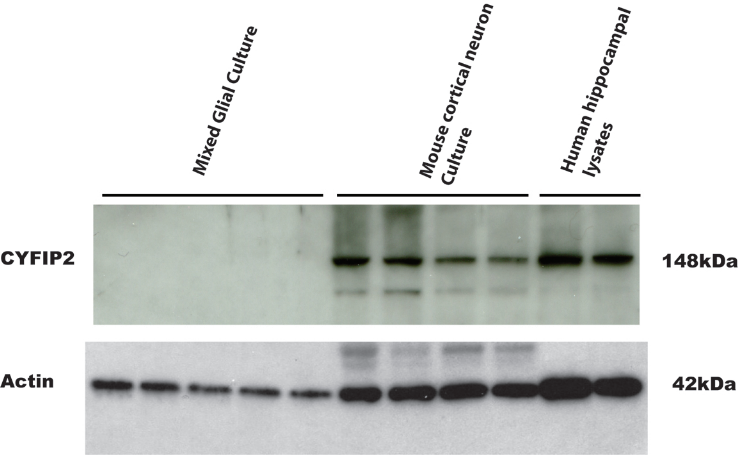
Mixed Glial Culture
Mouse cortical neuron Culture
Human hippocampal lysates
CYFIP2
148kDa
Actin
42kDa
