# Supplementary figures and images for: Alzheimer-related decrease in CYFIP2 links amyloid production to tau hyperphosphorylation and memory loss
Source: Brain. 2016 Aug 14;139(10):2751–65. doi: 10.1093/brain/aww205 (PMC5035822; doi:10.1093/brain/aww205)

### a) Sociability

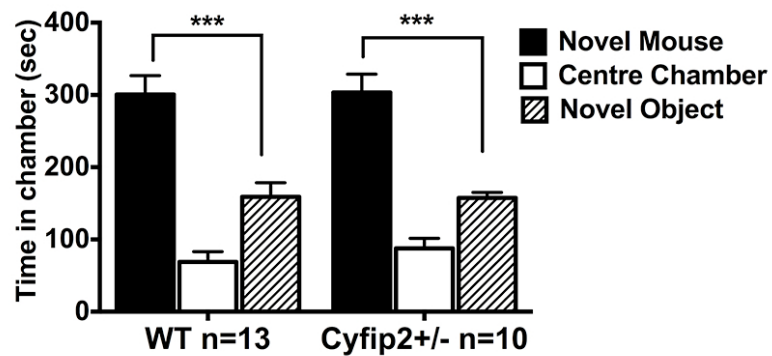

### b) Social Novelty

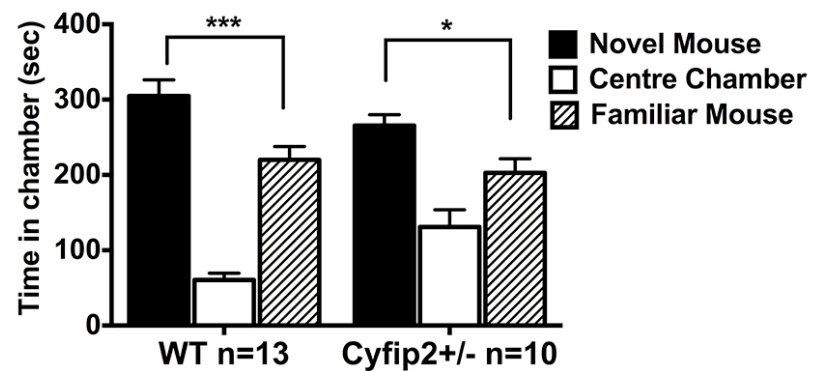

### c) Marble burying

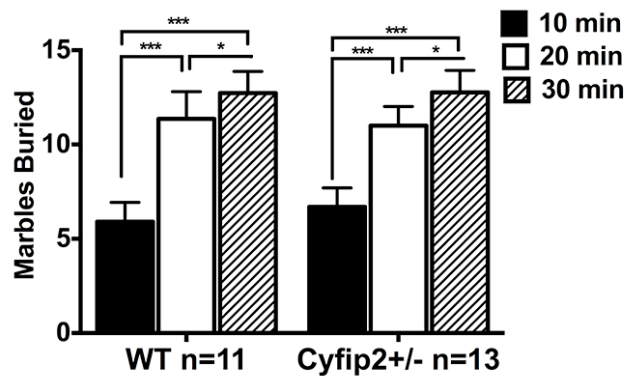

Supplement: Supplementary Data [file aww205_supplementary_data.zip › Supplemenatry figure 9.pdf]

# A CYFIP2

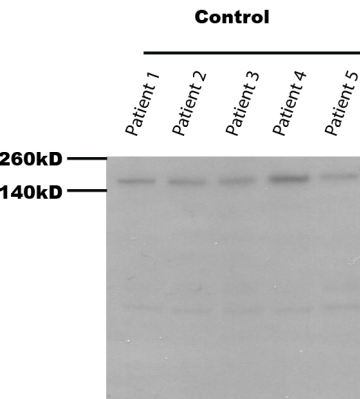

# B NSE

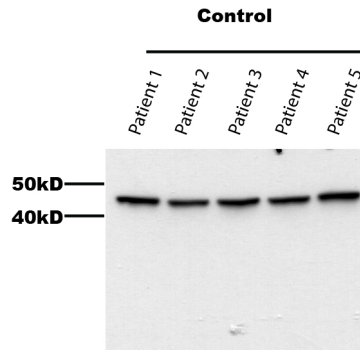

Supplement: Supplementary Data [file aww205_supplementary_data.zip › Supplementary Figure 1.pdf]

A)

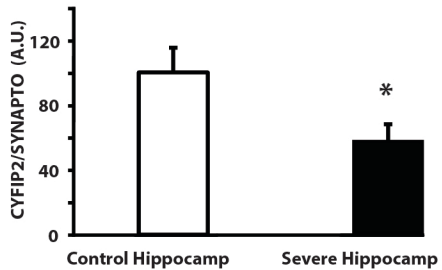

B)

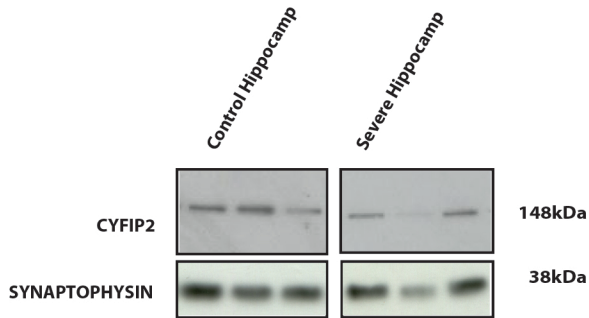

Supplement: Supplementary Data [file aww205_supplementary_data.zip › Supplementary Figure 3.pdf]

A)

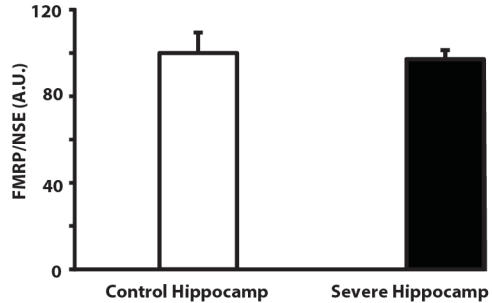

B)

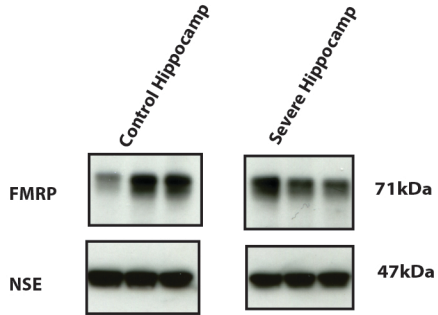

Supplement: Supplementary Data [file aww205_supplementary_data.zip › Supplementary Figure 4.pdf]

A)

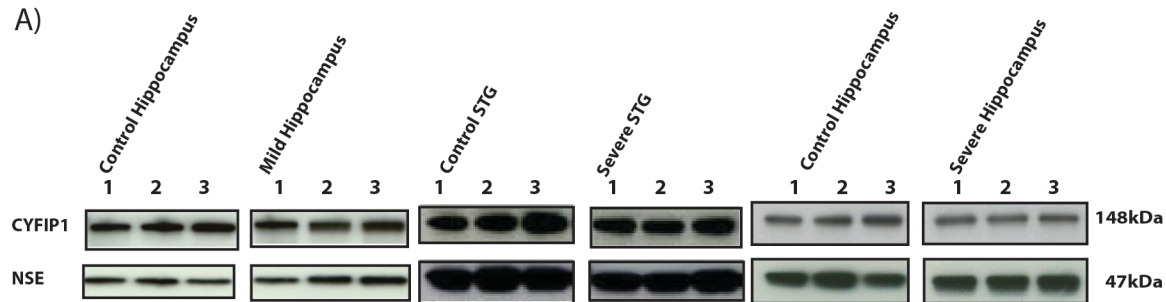

B)

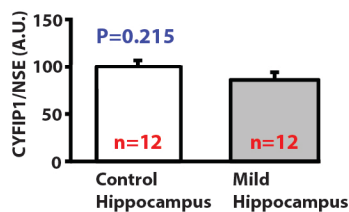

C)

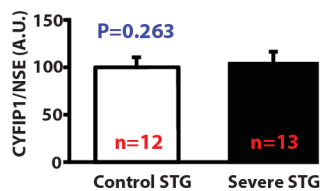

D)

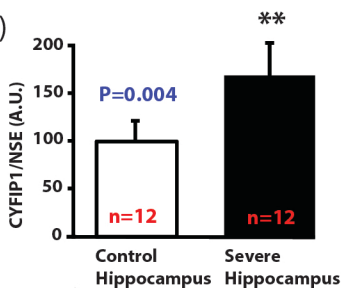

E)

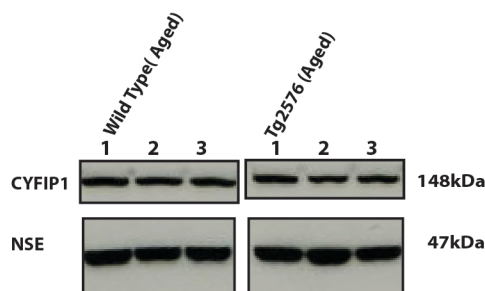

F)

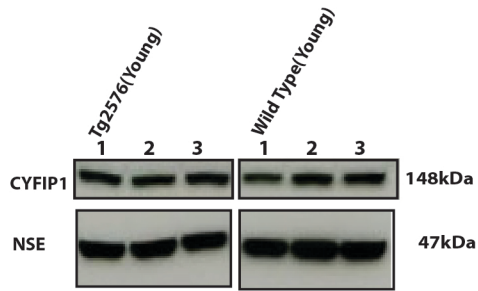

G)

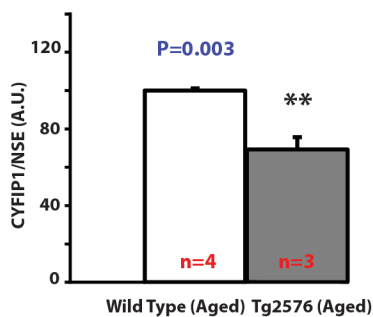

H)

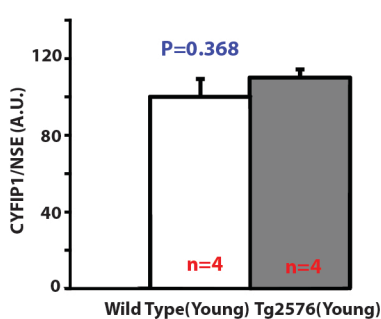

Supplement: Supplementary Data [file aww205_supplementary_data.zip › Supplementary Figure 5.pdf]

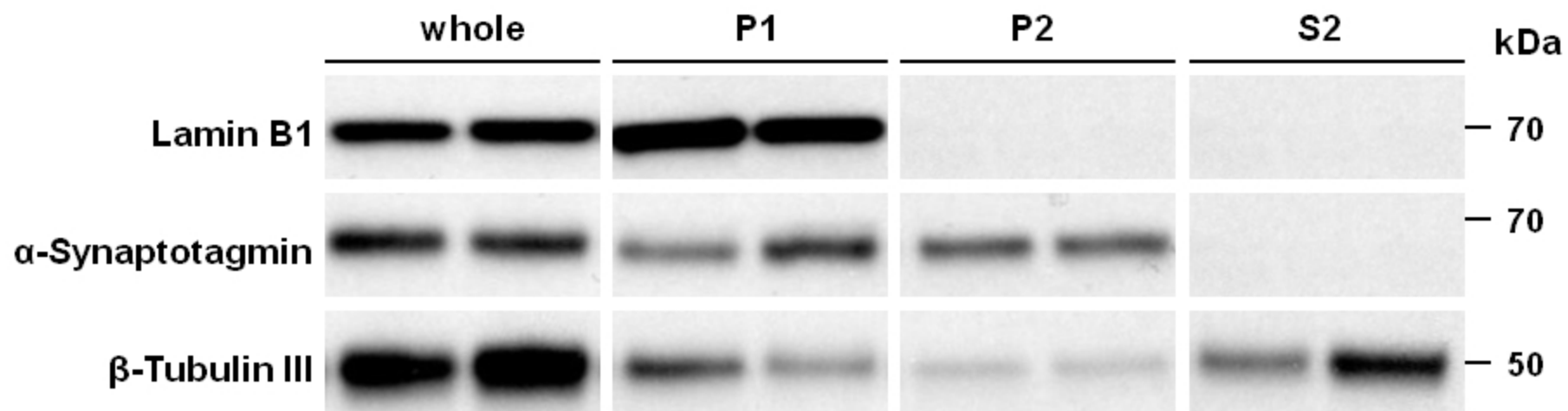

Supplement: Supplementary Data [file aww205_supplementary_data.zip › Supplementary figure 6.pdf]

**A**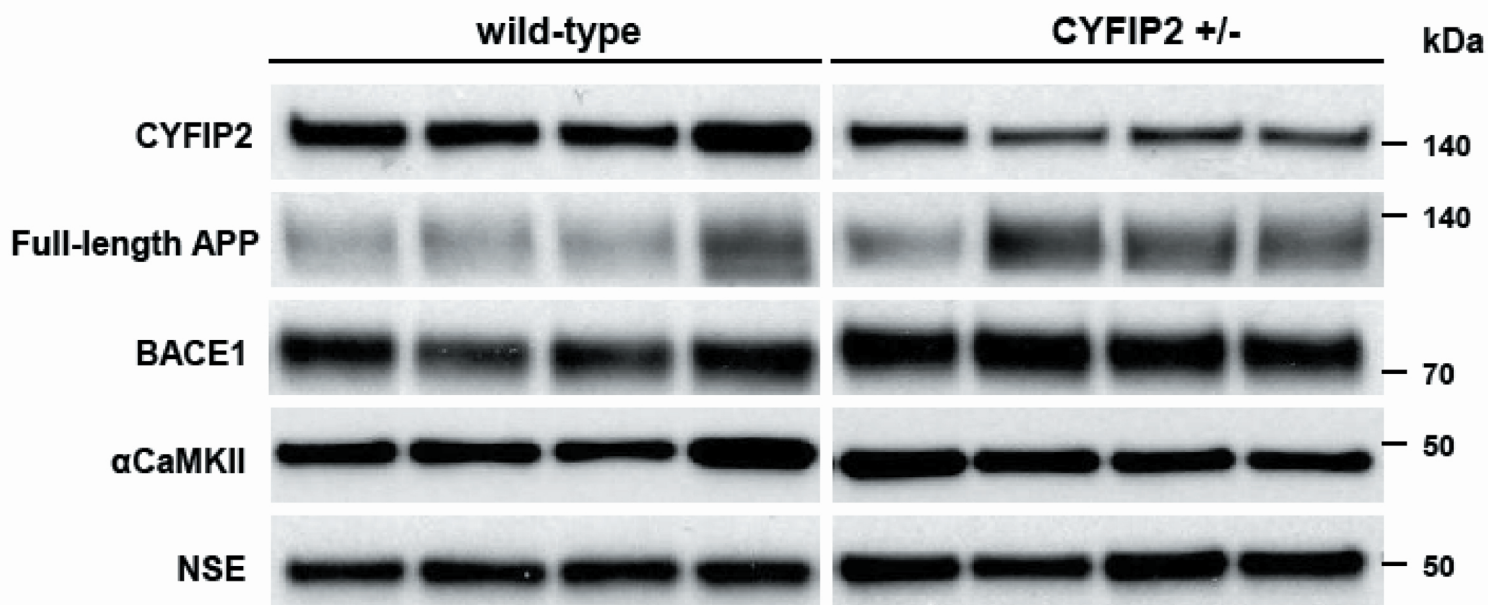**B**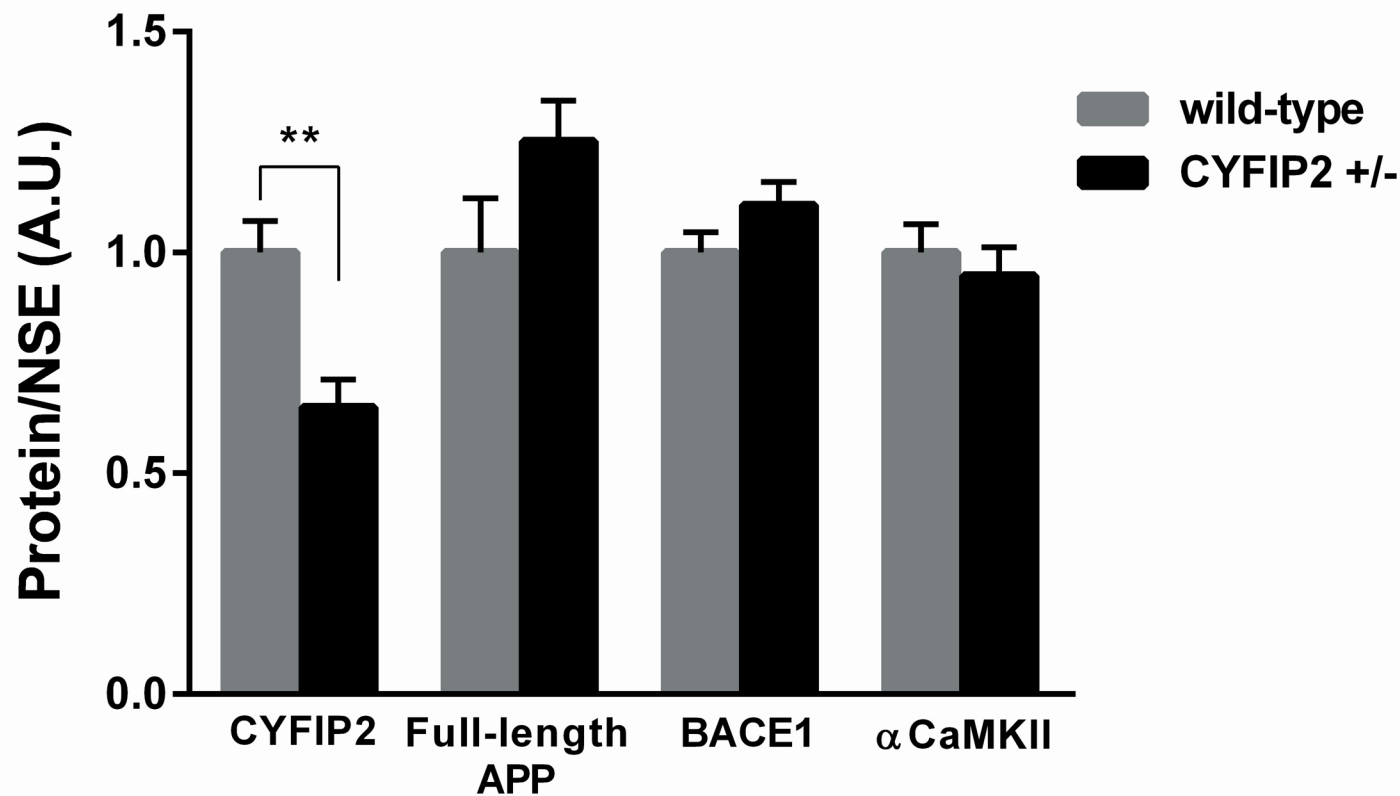

Supplement: Supplementary Data [file aww205_supplementary_data.zip › Supplementary figure 7.pdf]

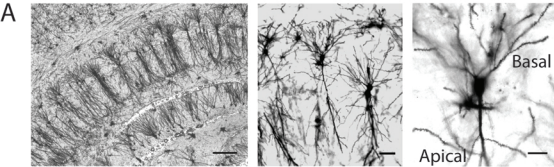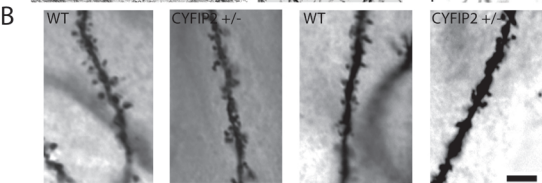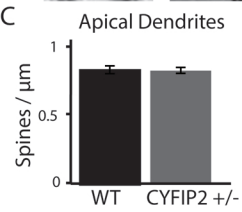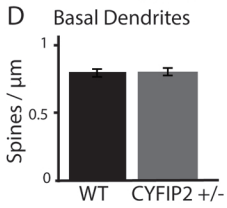

Supplement: Supplementary Data [file aww205_supplementary_data.zip › Supplementary figure 8.pdf]
